# Competition for refuelling rather than cyclic re-entry initiation evident in germinal centers

**DOI:** 10.1126/sciimmunol.abm0775

**Published:** 2022-03-11

**Authors:** Ziqi Long, Bethan Phillips, Daniel Radtke, Michael Meyer-Hermann, Oliver Bannard

**Affiliations:** 1 MRC Human Immunology Unit, Nuffield Department of Medicine, MRC Weatherall Institute of Molecular Medicine, University of Oxford, OX3 9DS, UK; 2 Department of Systems Biology and Braunschweig Integrated Center for Systems biology (BRICS), Helmholtz Center for Infection Research, Rebenring 56, D-38106 Braunschweig, Germany; 3 Institute for Biochemistry, Biotechnology and Bioinformatics, Technische Universitaet Braunschweig, Braunschweig, Germany

## Abstract

Antibody affinity maturation occurs in germinal centers (GCs) through iterative rounds of somatic hypermutation and proliferation in dark zones (DZs) and selection in light zones (LZs). GC B cells exit cell cycle a number of hours before entering LZs, therefore continued participation in responses requires they subsequently re-enter cell cycle and move back to DZs, a process known as cyclic re-entry. Affinity enhancements are thought to arise by B cells having to compete to initiate cyclic re-entry each time they enter LZs, with T cell help being determining, however direct proof is lacking. Using Fucci2 mice, we confirmed an association between B cell receptor affinity and the first step of cyclic re-entry, S phase initiation from a resting LZ state. However, neither T cell ablation nor MHCII deletion prevented resting LZ cells from re-entering cell cycle, and this late G1-S transition was also not detectably restricted by competition. In contrast, using BATF induction as exemplar, we found that T cells “refuelled” LZ cells in an affinity-dependent manner that was limited both by competition and cells’ intrinsic antigen-acquiring abilities. Therefore, cyclic re-entry initiation and B cell refuelling are independently regulated in GCs, which may contribute to permitting cells of different competencies to be sustained alongside each other and allow T cell support to be provided across a dynamic range commensurate with affinity. We speculate that this less binary selection mechanism could help GCs nurture complex antibody maturation pathways and support the clonal diversity required for countering fast evolving pathogens.

## Introduction

Antibody affinity maturation occurs in germinal centers (GCs) through iterative rounds of somatic hypermutation (SHM) and clonal selection (1–3). This generates bespoke antibodies that are more potent than their germline counterparts and can fill functional holes in antibody repertoires. GCs form in B cell follicles of draining secondary lymphoid tissues following infection or immunisation and quickly polarise to contain two distinct compartments, known as light zones (LZs) and dark zones (DZs), that GC B cells move back and forth between. The movement of B cells between zones is associated with changes in phenotypic and behavioural states (4, 5). As such, the distribution of tasks is separated, with SHM and proliferation occurring mostly in DZs and positive clonal selection in LZs (6–8).

The selection mechanisms that shepherd antibody affinity enhancements in GCs have been intensively researched. Popular models are based around the central concept, first proposed >25 years ago (7), that GC B cells must compete in an affinity dependent manner to undergo cyclic re-entry each time they enter LZs (1–3). Fast dividing DZ cells exit cell cycle a number of hours before entering LZs (6, 9, 10), and cyclic re-entry may be defined by resting LZ cells re-starting cell division (S phase entry) and subsequently moving back to DZs. The majority of cells (70-90% (11)) are thought to fail this selection checkpoint, and consequently apoptose (2, 3, 8, 12). As such, while GC B cells move bi-directionally, a strong DZ to LZ bias exists (4).

A key requirement of GC B cell selection is that relative B cell receptor (BCR) affinities for cognate antigen are measured and compared. This was originally suggested to involve direct competition for antigen access (7, 8), however imaging-based studies seemingly disproved this because prolonged GC B-follicular dendritic cell (FDC) contacts, such as those that would block antigen access, were not observed (9). Stable GC B cell-T cell interactions were found to be relatively rare, which instead suggested the possibility that competition occurs at this step (13). Higher affinity B cells are expected to capture more antigen from FDCs and thereby display more peptide-MHC class II (pMHCII) complexes to limiting numbers of follicular helper T (T_fh_) cells (14), which in turn may distract them from neighbouring B cells. This hypothesis was later supported by studies in which loading of GC B cells with exogenous peptide strongly promoted DZ accumulation and conferred a striking participation advantage (4). Collectively, these findings suggested that antibody affinity enhancements may be favoured by T_fh_ cells preferentially selecting the highest affinity B cells to undergo cyclic re-entry each time they enter LZs. We now also know that, among those cells making it back to DZs, higher affinity cells also receive a stronger dose of help which leads to higher or more sustained expression of key cell cycle regulators such as c-Myc and cyclin D3, which facilitates more and faster DZ cell division (15–19).

However, several concerns remain. A selection mechanism that is overly stringent towards affinity risks biasing antibody responses towards a small number of readily-targeted or highly abundant epitopes that will not necessarily always represent key sites of vulnerability. HIV provides a good example of this because primary repertoire antibodies usually do not bind conserved neutralizing sites with close to functional affinities, and extensive maturation is required (20). Moreover, pathogens will often escape neutralizing responses unless multiple distinct sites are targeted in concert, as is evident for SARS-CoV-2 (21). A such, the preferential expansion of high affinity B cells should not come at the expense of clonal diversity (3). Direct proof that GCs are indeed permissive to differing affinity B cells was recently provided when antibodies derived from single GC B cells were compared (22, 23). Such observations are hard to explain using models where most (lower affinity) cells are cleared from responses each time they enter LZs.

Prompted by these concerns, we aimed to test key assumptions of cyclic re-entry models. The first step of the cyclic re-entry process is the initiation of S phase by resting G1 LZ cells (herein referred to as cyclic re-entry initiation), therefore we assessed what inputs cells need for this to occur. Surprisingly, our findings exclude T cell help as being acutely limiting, or even necessary, for driving this event because it still occurred both when they were ablated from ongoing responses as well as when MHCII was inducibly deleted from GC B cells. These findings led us to ask whether this step is instead restricted by competition for other limiting factors such as antigen access, but we also failed to find evidence for that. Instead, we found that cyclic re-entry initiation and GC B cell metabolic refuelling, i.e., the induction of biochemical pathways required for DZ maintenance and that control cell division capacity, are independently regulated, with the latter being provided by T cells in an affinity-dependent manner that is restricted by both competition and a cell’s intrinsic ability to acquire antigen. We propose that this less binary selection mechanism contributes to allowing both fast and slow maturing antibodies to evolve alongside each other without excessively repressing each other’s potential.

## Results

### Cyclic re-entry initiation can occur independently of cognate T cell interactions

**Figure 1:**
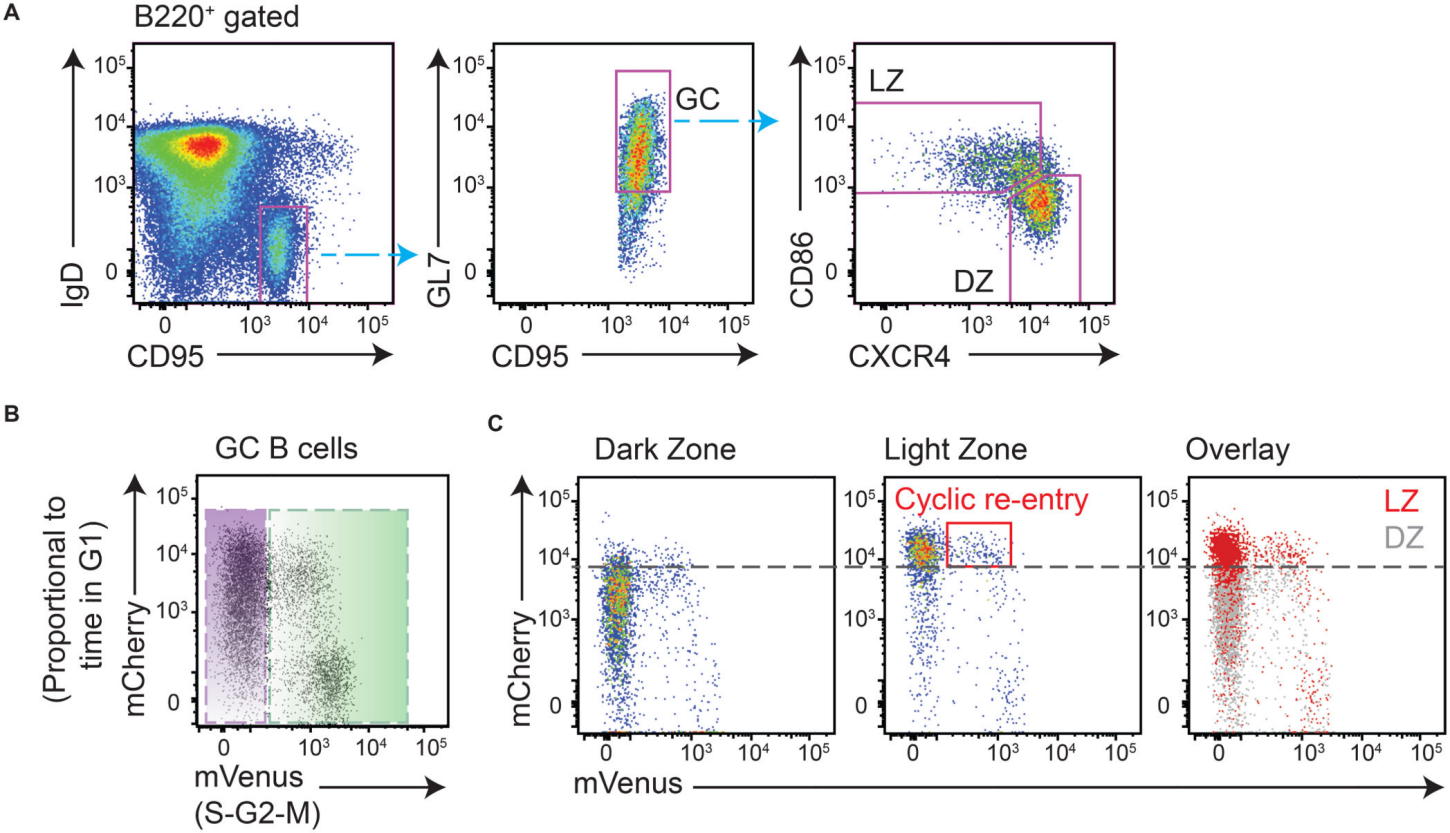
Identification of light zone cells initiating cyclic re-entry in Fucci2 mice. Analysis of splenic responses in Fucci2 mice on day 8 post-SRBC immunisation. (A) GC B cell DZ and LZ populations identified as shown. (B) G1 cells and S-G2-M phase cells are identified. mCherry levels accumulate with time post-mitosis, and mVenus accumulates with time in S phase; as such, early S phase cells are mCherry^high^ mVenus^low^. (C) LZ cells are mostly resting and therefore have higher mCherry levels than DZ cells. Dashed line provided to aid comparison. LZ cells initiating cyclic re-entry (early S phase) are identified by the red box.

To test the assumption that T cells are essential and limiting for cyclic re-entry initiation by LZ cells, approaches were established for tracking this event in vivo and for temporally perturbing B-T interactions. Cyclic re-entry is defined by LZ cells entering back into cell cycle from a resting late G1 state and subsequently moving back to the DZ phase, however we focussed only on the first and most tractable of these steps; cyclic re-entry “initiation”. R26p-Fucci2 mice carry a constitutively expressed bicistronic transgene consisting of mVenus-hGem(1/1 10) and mCherry-hCdt1(30/120) (24). Cells from these mice start accumulating mVenus protein as they enter S phase, with it then being rapidly degraded at mitosis (Fig. 1A, B). Post-mitotic cells start their lives as double negative (mVenus^neg^ mCherry^neg^), but accumulate mCherry protein from thereon until the G1-S checkpoint. mCherry degradation then begins, with completion by mid-S. Consequently, it is possible to identify both a cell’s cell cycle stage and the relative time it spent in that phase. The fidelity of this model for GC B cells was demonstrated previously (10), and confirmed here (Fig. S1A–C); EdU incorporation is restricted to mVenus^+^ S phase cells, while post-mitotic cells become brighter for mCherry with time post-EdU labelling due to protein accumulation during G1. Following mitosis, DZ cells either re-enter S phase a relatively short period later and remain in that zone (10, 15), or they rest for longer periods then downregulate CXCR4 and transition to their LZ state (~8hrs after S phase) (6, 10). Therefore, resting LZ cells display higher mCherry levels than DZ cells (Fig. 1C). LZ cells initiating cyclic re-entry in Fucci2 mice are identifiable by their entering early S phase (mVenus^low^) from a late G1 stage (mCherry^high^). Cyclic re-entry initiation is distinct from “inertial” S phase entry in DZs (25), which occurs at earlier G1 stages.

**Figure 2:**
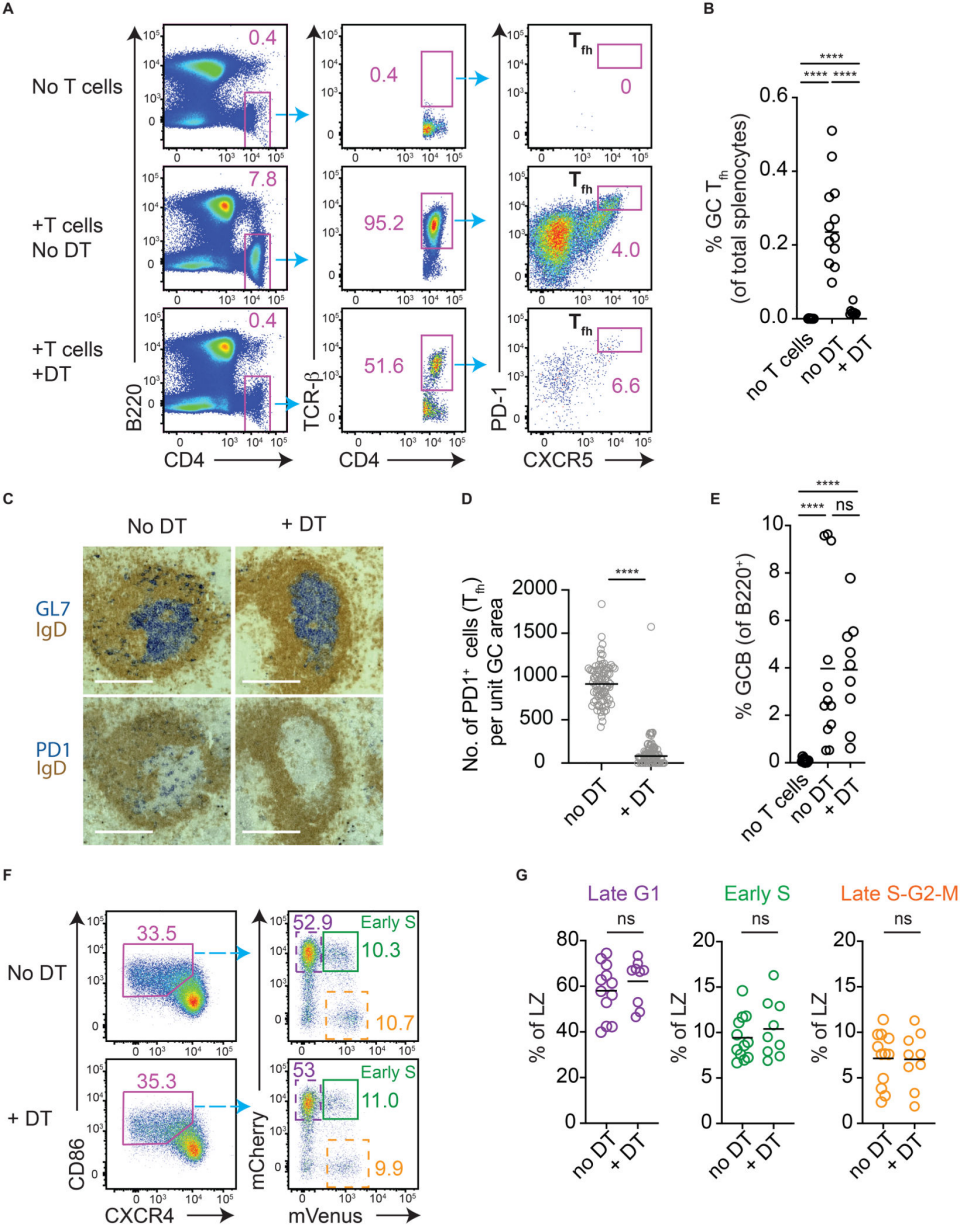
Cyclic re-entry initiation is not acutely limited by GC T_fh_ cell availability. Analysis of splenic responses in R26-Fucci2 *Tcrb/d^-/-^* mice that received an adoptive transfer of CD4-Cre Rosa-DTR T cells and SRBC/LPS immunisation. Analysis is on days 8 and 10, 48hrs post- DT (or vehicle) treatment. (A) GC T_fh_ cells were gated as shown, (B) and quantitated. (C) The relative absence of PD1^+^ T_fh_ cells in GL7^+^ GCs was determined by IHC (scale bar, 220μm), (D) and enumerated. Each point is a single GC, pooled from 5-6 mice/condition. (E) Frequencies of GC B cells (IgD^low^ CD95^+^ GL7^+^). (F) LZ populations were identified (left, means +/- S.E.M.; no DT 33% +/- 0.9, +DT 35% +/- 1.1) and frequencies of early S phase cells (Green), as well as late G1 (dashed purple), late S-G2-M (dashed orange), determined (right). (G) Results from pooled experiments with means. Results are representative of (A, F), or pooled from (B, E, G), 5 experiments each with 1-3 mice per condition. Analysis, Mann-Whitney U test throughout except (E, no DT vs DT, G) unpaired student’s T test. ****p < 0.0001

Fucci2 mice were bred onto a T cell-deficient background (R26-Fucci2 *Tcrb/d^-/-^*mice). While GCs do not usually develop in such animals, their establishment could be supported by the adoptive transfer of T cells prior to immunisation with sheep red blood cells (SRBCs) adjuvanted with lipopolysaccharide (LPS) (Fig. 2A-C). The transferred T cells expressed a diphtheria toxin receptor (DTR) (from CD4-Cre^+^ R26-loxp-stp-loxp-DTR mice), thereby allowing them to be efficiently and specifically eliminated from established responses (Fig. 2A-D). T cell ablation is maximal within <24hrs of diphtheria toxin (DT) treatment (26), therefore GC B cell responses were analysed at 48hrs. This adoptive transfer model suffered from variable response magnitudes which may mask subtle differences, however there was no detectable change in GC B cell frequencies by this time point (Fig. 2E). Despite a paucity of T_fh_ cells, LZs in DT treated animals contained very clear populations of cells initiating S phase from a late G1 state, with no detectable differences relative to no DT controls (Green gates/plots, Fig. 2F, G), and normal frequencies reaching S-G2-M stages while still in that zone (Dashed orange).

**Figure 3:**
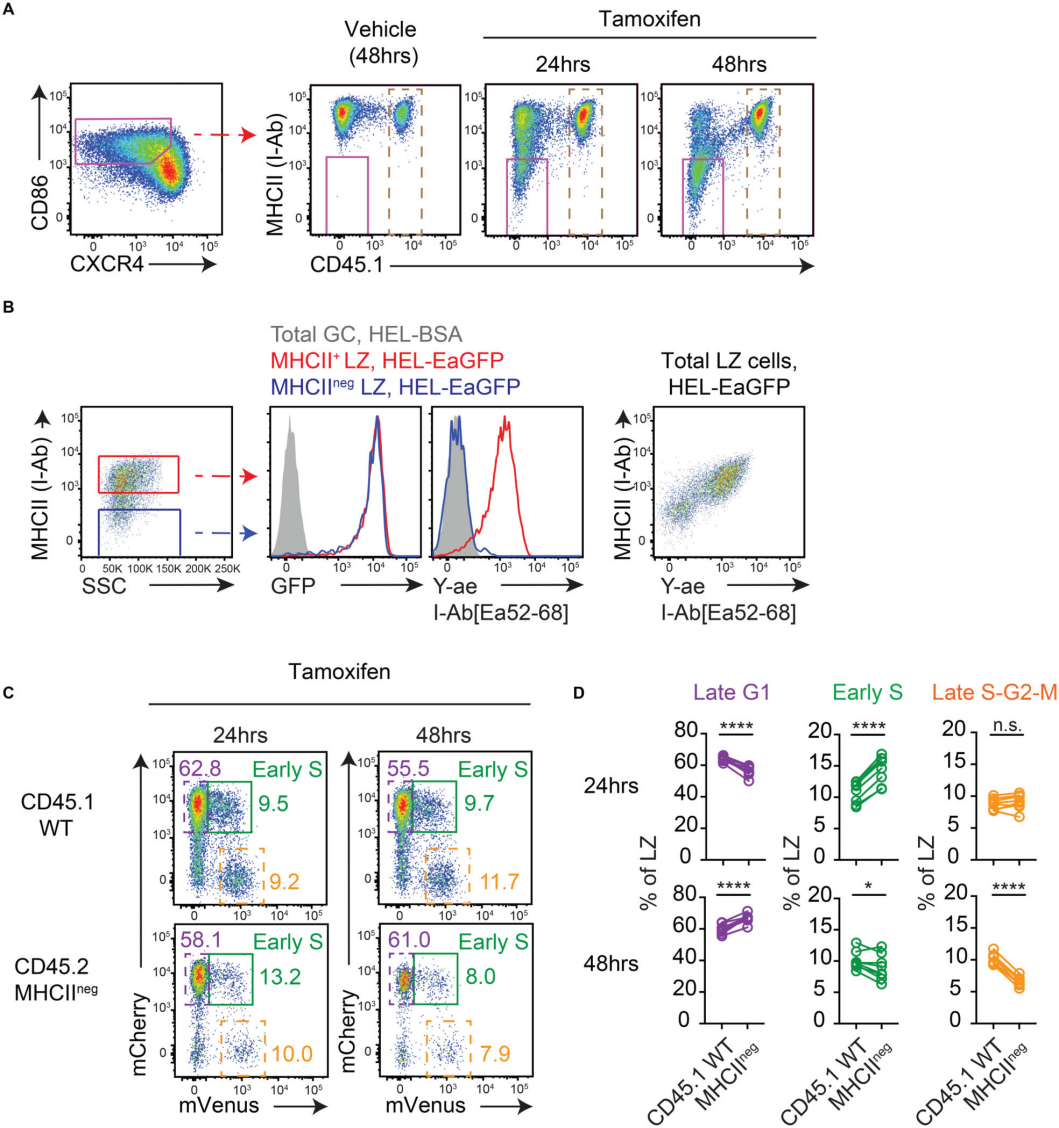
Cyclic re-entry initiation does not require MHCII presentation. (A) Analysis of WT:R26-CreERT2 MHCII^fl/fl^ Fucci2 mixed BM chimeras on day 8 post-SRBC immunisation, at indicated times after tamoxifen treatment. Representative plots of MHCII deletion by LZ splenic GC B cells (IgD^low^ CD95+ GL7^+^). Dashed gates identify WT cells used in (C-D). (B) Antigen acquisition (GFP) and pMHCII presentation (Y-ae mAb staining) by day 8 SW_HEL_ *Cd79a-CreERT2* MHCII^fl/fl^ LZ GC B cells (40hrs post-tamoxifen) following 2 hr *ex vivo* incubations with HEL-EaGFP or HEL-BSA (control).(C) LZ populations gated in A. Frequencies of late G1 (dashed purple), early S (green) and late S-G2-M (dashed orange) cells determined for WT and MHCII-deleted populations, (D) and summarised. Lines join populations from individual mice. Results show representative FACs plots (A, B, C) or pooled results (D) from 2 experiments each containing 4-5 mice per condition. Analysis, paired two-tailed Student’s t test. *p < 0.05, ****p < 0.0001.

T cell ablation was efficient but not absolute, and certain regulatory inputs may be lacking or abnormal in T cell-deficient GCs. Therefore, to corroborate these findings, an approach for deleting MHCII from subsets of GC B cells during ongoing responses was established. To this end, we reconstituted lethally irradiated mice with bone marrow (BM) cells expressing a tamoxifen regulatable Cre, “floxed” MHCII (I-Ab, *H2-Ab1*) alleles (hereafter referred to as MHCII^fl/fl^) and the Fucci2 reporter (R26-CreERT2^+^ MHCII^fl/fl^ Fucci2 CD45.2) alongside “WT” Fucci2 CD45.1 control cells at a ratio of ~60:40 (27, 28). Tamoxifen treatment of SRBC immunised chimeric mice led to rapid deletion of MHCII from a fraction of splenic GC B cells within <24hrs (Fig. 3A), consistent with expectations (29). The inability of MHCII-deleted GC B cells to present peptide antigen was confirmed by performing similar MHCII-deletion experiments but using HEL-specific SW_HEL_ GC B cells (*Cd79a-CreERT2^+^* MHCII^fl/fl^ B cells, transferred into WT hosts and immunized with HEL3x-SRBCs/LPS) and incubating them *ex vivo* with HEL-EaGFP (containing Ea52-68 peptide) or HEL-BSA conjugates for 2hrs prior to staining with the I-Ab[Ea52-68] complex-specific Y-ae monoclonal antibody (Fig. 3B) (30), as well as by assessing activation marker expression by OVA-specific OT-II T cells after co-culture with FACS sorted polyclonal MHCII-deleted (and control) LZ cells loaded with exogenous cognate peptide antigen (Fig. S2A).

We next assessed cyclic re-entry initiation by MHCII-deleted cells in the immunised mixed BM chimeras, with comparisons made to paired WT cells. At 24hrs post-tamoxifen, no reductions in the proportions of cells initiating S phase were observed for MHCII-deleted LZ cells (Fig. 3C, D, green gates/points); there was in fact a small increase, which probably reflects MHCII being more stable in certain quiescent subsets such as pre-memory cells that reside in LZs for longer (31–33). By 48hrs, modest reductions in cyclic re-entry initiation by MHCII-deleted LZ cells were evident, with slightly larger impacts seen at late S-G2-M phases (orange gates/points). This difference relative to the non-competitive T cell ablation setting, where no defect was seen (Fig. 2), may reflect the better sensitivity and/or competitive nature of this assay, the kinetics post-intervention, or differences in the regulatory environment. Possible explanations for the larger effect at late cell cycle stages include changes in the LZ-DZ transition rate and/or differences in cell cycle progression. Despite these issues, it was striking that large fractions (seemingly the majority of normal numbers) of MHCII-deleted LZ cells continued to initiate S phase despite lacking the essential machinery for cognate B-T interactions (and while being surrounded by MHCII^+^ competitors). Cyclic re-entry initiation was even still evident at days 4 and 6 post-tamoxifen (Fig. S2B). Consistent results were obtained using alternative methods for identifying early S phase cells (temporally separated EdU/BrdU double labelling, Fig. S2C, D), as well as following immunisation with a different immunogen (NP-KLH/alum, 2.5 day post-tamoxifen time point) using EdU to identify total S phase cells (Fig. S2E), thereby confirming that the effect was not specific for the Fucci2 reporter, the time points or the immunisation type. Additionally, back-gating confirmed that MHCII-deleted early S phase cells were bona-fide LZ cells rather than contaminating DZ cells (Fig. S2F); this conclusion was also supported by “cyclic re-entry” cells having LZ-like mCherry intensities (Fig. 3C). Finally, the unlikely possibility that these effects were caused by mismatched I-Aa and I-Eb heterodimer expression (the H2-Ea gene, encoding I-Ea, is inactive in 129X1/SvJ mice from which the MHCII locus derives)(34), was excluded by assessing cyclic re-entry initiation by MHCII-deleted SWHEL LZ cells that were supported by OT-II CD4^+^ T cells (I-Ab-OVA223-239-restricted) in Tcrb/d^-/-^ hosts (Fig. S2G).

A requirement for T cell help for sustaining GC responses is well recognized and this was clearly evident here by tracking frequencies of CD45.2 cells that were MHCII-deleted at the different time points (Fig. S2H, I), alongside participation in the GC (Fig. S2J). Most MHCII deletion (~60%) occurred within the first 1.5 days of tamoxifen treatment (Fig. S2I). Detectable reductions in participation by CD45.2 cells were first apparent at 2 days post-tamoxifen, with major losses occurring after this (Fig. S2J). CreERT2 activity alone caused some loss of GC B cells but this was not the major cause of the GC participation defects (Fig. S2K). The asynchronous kinetics of MHCII losses hindered our efforts to determine with any precision how long “unhelped” cells persist, however the slight reductions in participation that were evident at 48hrs suggest that the periods were probably not more than 1-3 days for most cells (although very small MHCII-deleted populations remained even at 6 days post-tamoxifen).

Together, these results demonstrate that, while cognate B-T interactions are essential for sustaining prolonged participation in GCs, they are not acutely required during a particular zonal visit for LZ B cells to re-enter cell cycle from their resting state, a defining feature of cyclic re-entry initiation.

### Cyclic re-entry initiation is not restricted by competition

**Figure 4:**
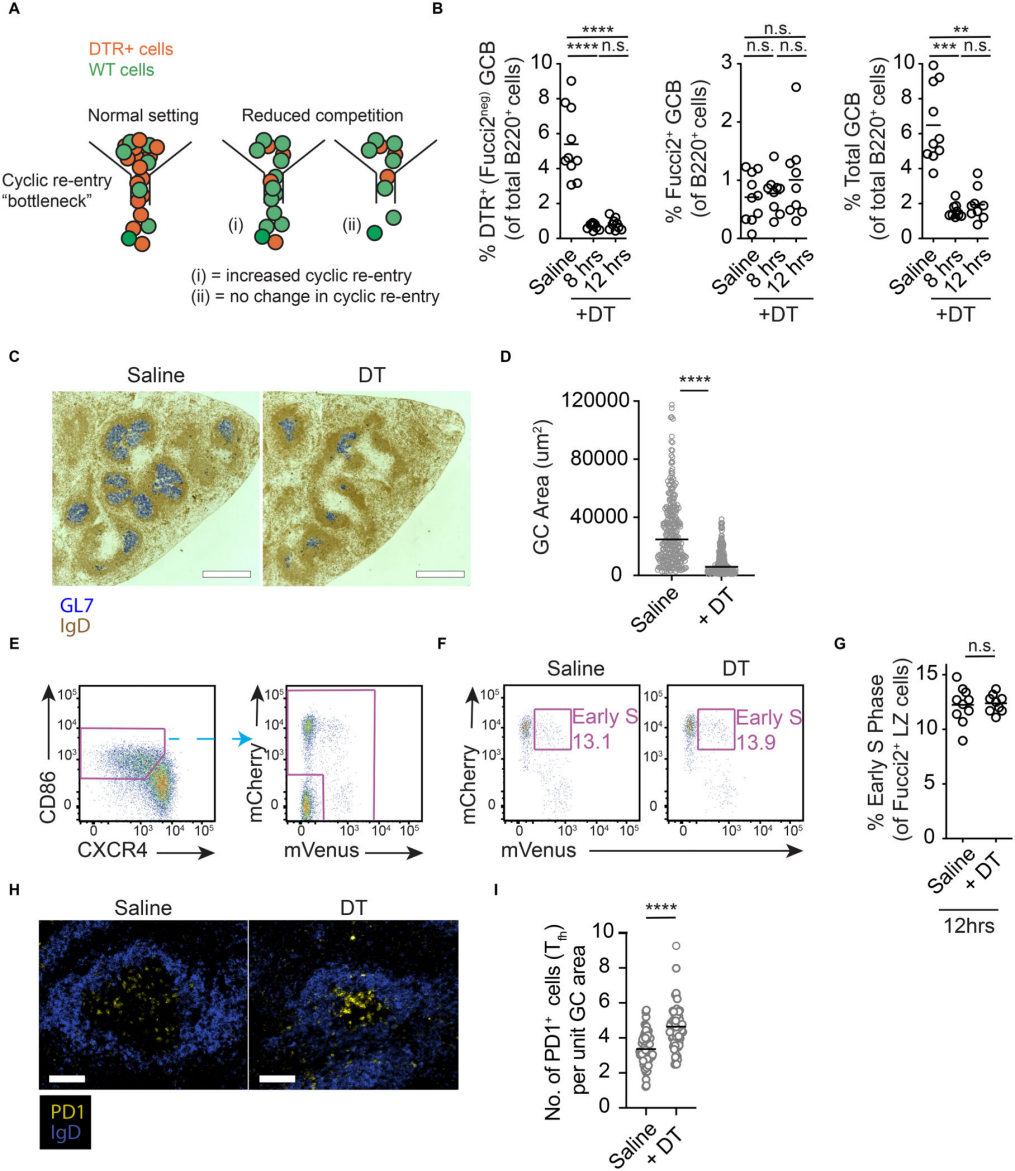
No detectable role for competition in determining cyclic re-entry initiation. (A) Possible outcomes after competition is relieved in LZs. Models assuming LZ B cells compete for initiating cyclic re-entry (left) predict the fraction of remaining cells (green) entering S phase should increase when competing cells (orange) are removed (middle). Alternatively, if the process is not acutely competitive, cyclic re-entry initiation rates would not change (right). (B) GC B cell analysis after partial DT-mediated GC ablation in AID-DTR:Fucci2 (80:20) mixed BM chimeric mice, on day 8 post-SRBC immunisation. Time points post-DT treatment are indicated, vehicle at 8 hrs. Frequencies of DTR^+^ (left), Fucci2^+^ (middle), and total (right) GC B cells. (C) Representative splenic IHC sections (scale bar, 550μm) and (D) quantitation of GC areas, at 12hrs. (E) Fucci2^+^ LZ cells were gated and (F, G) frequencies of early S cells enumerated. (H, I) PD1^+^ cells per unit GC area were quantified by immunofluorescence staining at 15hrs post-DT (scale bar, 100μm). Points in D, I represent single GCs. G is pooled from 3 experiments, with 2 mice examined/group/experiment. FACS plots are representative of, and summaries are pooled data from, 2 experiments each containing 5 mice per condition. Analysis, (B, left, center) one-way ANOVA with Tukey’s multiple comparisons, (B right) Kruskal-Wallis with Dunn’s multiple comparisons, (D, I) Mann-Whitney, (G) unpaired two-tailed Student’s t test. **p <0.01, ***p < 0.001, ****p < 0.0001.

These surprising findings led us to consider whether cyclic re-entry initiation may depend on GC B cells acutely competing for unknown factors that are not T cell derived, e.g., factors provided by FDCs, or access to antigen. To this end, we tested the role of competition using an approach that was not biased for any single factor, by temporally removing a large fraction of the competing cells from LZs. This was achieved using mixed BM chimeric mice containing a majority of cells expressing a GC-restricted DTR (*Aicda^wt/cre^* R26-loxp-stp-loxp-DTR mice), and a smaller fraction of cells from Fucci2 donors (DTR-negative). If initiating cyclic re-entry is restricted by competition, rates of cyclic re-entry initiation should increase when competition is removed (Fig. 4A). Alternatively, a finding that cyclic re-entry initiation rates do not change would suggest that this process is not acutely limited by cells successfully competing for selection cues.

Delivery of DT to chimeric mice at day 7 post-immunisation caused the elimination of large fractions of the DTR^+^ population within 8hrs, with no significant further loss occurring later (Fig. 4B, left plot). Overall GC B cell frequencies decreased from ~6% of B220^+^ cells to <2%, therefore ~2/3 of the competition was relieved (Fig. 4B, right plot). Importantly, the effect was specific for DTR^+^ GC B cells because the frequencies of DTR^neg^ (Fucci2^+^) GC B cells was not impacted (Fig. 4B, center plot). Quantitation of GC areas confirmed an overall reduction in GC sizes as opposed to some GCs being preferentially lost (Fig. 4C, D).

A short rest period following DT treatment was provided for potential competition-restricted selection to occur, then cyclic re-entry initiation was measured at 12hrs. Despite facing only ~1/3 the normal competitors, LZ cells were no more likely to initiate cyclic re-entry in DT treated animals (Figs. 4E-G). Similar results were obtained at a slightly later time-point (15hrs) when the 30-minute BrdU-labelling approach was used for identifying S phase cells (Fig. S3A-C). As such, the presence of competition was not acutely restraining this aspect of cyclic re-entry initiation. The partial ablation of GC B cell populations caused increases in the GC T_fh_:B cell ratios (Fig. S3D-F, with GC-type T_fh_ defined by higher CXCR5 and PD1 staining intensity), and led to a higher density of PD1^+^ cells within GCs (Fig. 4H-I). As such, these results also corroborate those from Figs. 2, 3 in terms of T cell numbers not being acutely limiting for S phase initiation. Finally, we also confirmed that LZ cells do not show evidence of competing directly for antigen access because no detectable increases in Nur77-GFP accumulation were evident when using Nur77-GFP cells (35), alongside DT-expressing cells (Fig. S3G, H).

### High BCR affinity is associated with cell cycle initiation in GC light and dark zones

Cyclic re-entry models predict that high affinity cells should be most prevalent within LZ populations that are initiating cyclic re-entry. Our findings had revealed cyclic re-entry initiation to be relatively insensitive to acute changes in T cell numbers, MHCII deletion and to competition, however it remained possible that it occurs when cells reach certain BCR signaling thresholds and that this favors high affinity cells. We therefore tested whether an association between BCR affinity and cyclic re-entry initiation exists. Small populations of SW_HEL_ Fucci2 B cells were transferred into WT recipients that were subsequently immunised with SRBCs decorated with recombinant HEL^3x^. HEL^3x^ is a modified HEL protein to which SW_HEL_ B cells affinity mature in GCs (~80-fold affinity increases through acquisition of a Y53D mutation) (36). Although heterozygous SW_HEL_ donor mice contain both HEL-specific and non-specific B cells, using this antigen-specific immunization regimen only HEL-specific cells enter GCs. Therefore, SW_HEL_ GC B cells that have improved their affinities through SHM can be identified by staining with fluorescently labelled HEL^3x^.

**Figure 5:**
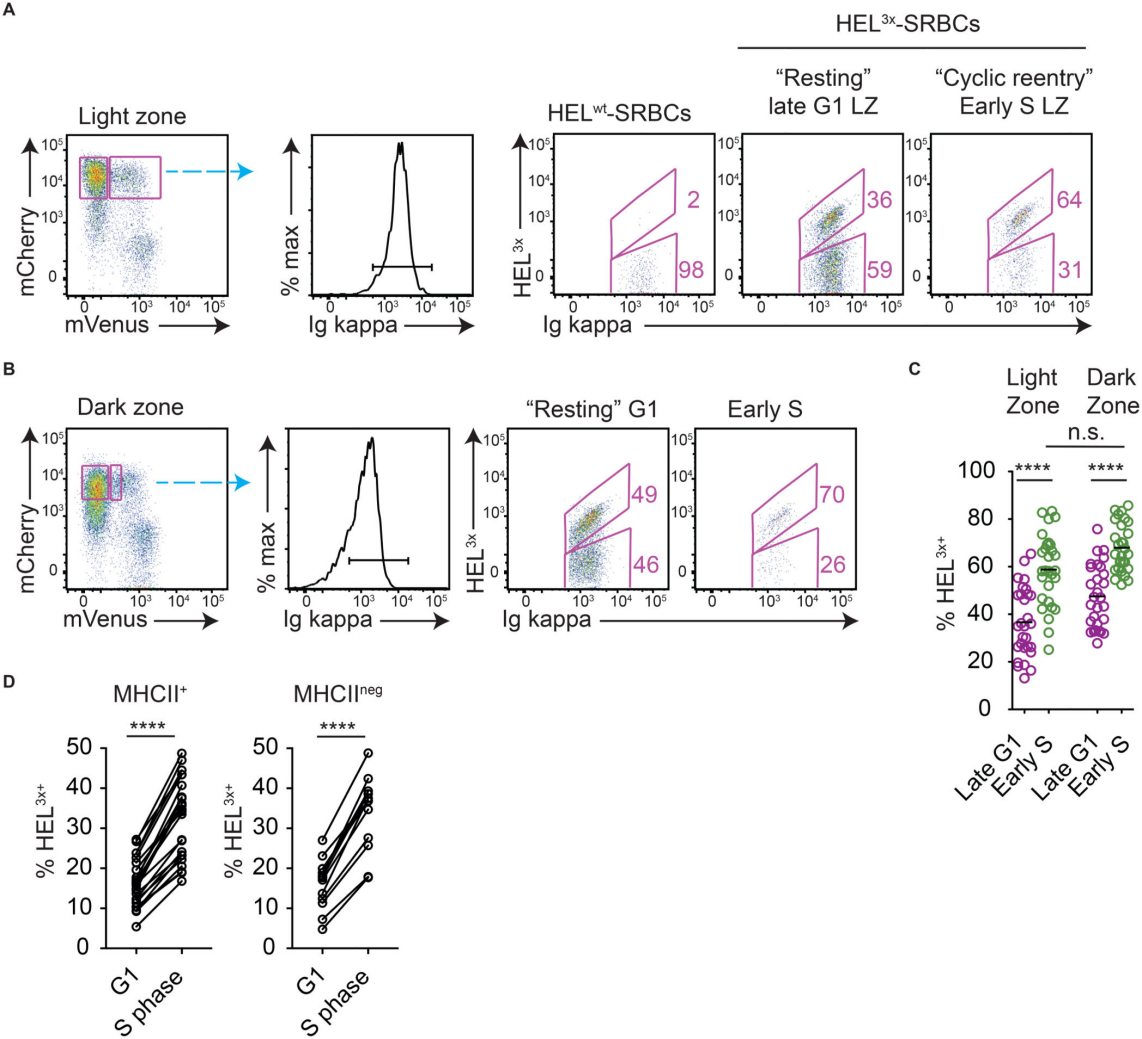
Cyclic re-entry initiation is associated with BCR affinity. Analysis of splenic SW_HEL_ Fucci2 GC B cells responding to HEL3x-SRBC/LPS (or HEL^WT^-SRBCs, staining control) immunization, on day 8 or 9. (A) The frequencies of HEL^3x^-bright cells within BCR(Igk)^+^ SW_HEL_ LZ (CXCR4^low^ CD86^+^ IgD^low^ GL7^+^ CD45.1^+^) populations were determined for each cell cycle stage. (B) Similar analysis was performed for DZ (CXCR4^high^ CD86^low^) cells. (C) Summary of results pooled from 7 experiments each with 3-5 mice. (D) Similar experiments with SW_HEL_ *Cd79a-CreERT2* MHCII^fl/fl^ B cells, where MHCII could be deleted (42hrs post-tamoxifen). Mice received EdU 30mins before analysis to mark S phase cells. G1 (EdU^neg^) and S phase (EdU^+^) populations were gated, for MHCII^+^ and MHCII-deleted LZ cells, and frequencies of HEL^3X-^bright cells determined. Results pooled from 3 experiments each with 2-10 mice/group. Lines join populations from single mice. MHCII^+^ populations include tamoxifen and vehicle mice. Frequencies in (A, B) are from representative mice. Analysis, (C) RM two-way ANOVA with multiple comparisons, (D) paired two-tailed Student’s t test. ****p < 0.0001.

The frequencies of late G1 and early S phase LZ SW_HEL_ cells that were HEL^3x^-bright (high affinity) were determined on days 8 or 9 post-immunisation (Fig. 5A). Mice immunised with HEL^wt^-SRBCs provided HEL^3x^ staining controls. HEL^3x^-bright cells were enriched within the early S phase LZ population, confirming that high affinity cells are more likely to initiate cyclic re-entry. However, this effect was not unique to the LZ; in fact, similar or marginally greater (non-significant) affinity enrichments were observed within subsets of DZ cells that were entering S phase (Fig. 5B-C). Therefore, the association between BCR affinity and cyclic re-entry initiation may reflect the cells being in a more actively dividing state rather than them having been preferentially recently selected.

Finally, we also explored the possibility that, while T cell interactions may not be acutely required for initiating cyclic re-entry, they might still be directly responsible and necessary for the higher affinity cells being favored at this step. However, similar associations between affinity and cyclic re-entry initiation were evident irrespective of whether the LZ cells examined were MHCII+ or MHCII-deleted when similar experiments were performed using SW_HEL_ *Cd79a-CreERT2* MHCII^fl/fl^ B cells (Fig. 5D), seemingly excluding that direct instruction by T cells was the sole cause of the enrichment.

In summary these results support that high affinity B cells are more likely to initiate cyclic re-entry from LZs, but also demonstrate that this does not directly depend upon them preferentially being selected by T cells during a given LZ visit.

### Light zone GC B cells compete for T cell derived refuelling cues

Our findings were not readily explained by simple T cell-dependent cyclic re-entry-based models for affinity maturation, therefore we explored other possible mechanisms. Beyond their previously assumed role in controlling cyclic re-entry initiation by LZ cells, T cells can determine DZ GC B cell division capacity depending upon the dose of help afforded (15–19). However, one conceptual concern is that it may be difficult for T cells to dose help across a dynamic range if cyclic re-entry initiation is triggered through the same pathways when cells reach a stringent, competitive signaling threshold. One might predict this would cause all cells to return to DZs with the same or similar division capacities. Our new findings provided a possible solution to this problem, therefore we investigated whether T cells may drive antibody affinity maturation by preferentially metabolically “refuelling” B cells (i.e., by inducing pathways required for DZ sustenance and controlling proliferative capacity), rather than being necessary and limiting for cyclic re-entry initiation. To start, we asked whether B cells demonstrate evidence of restricting each other’s access to refuelling from T cells at physiological presentation levels, a key requirement of competition.

**Figure 6:**
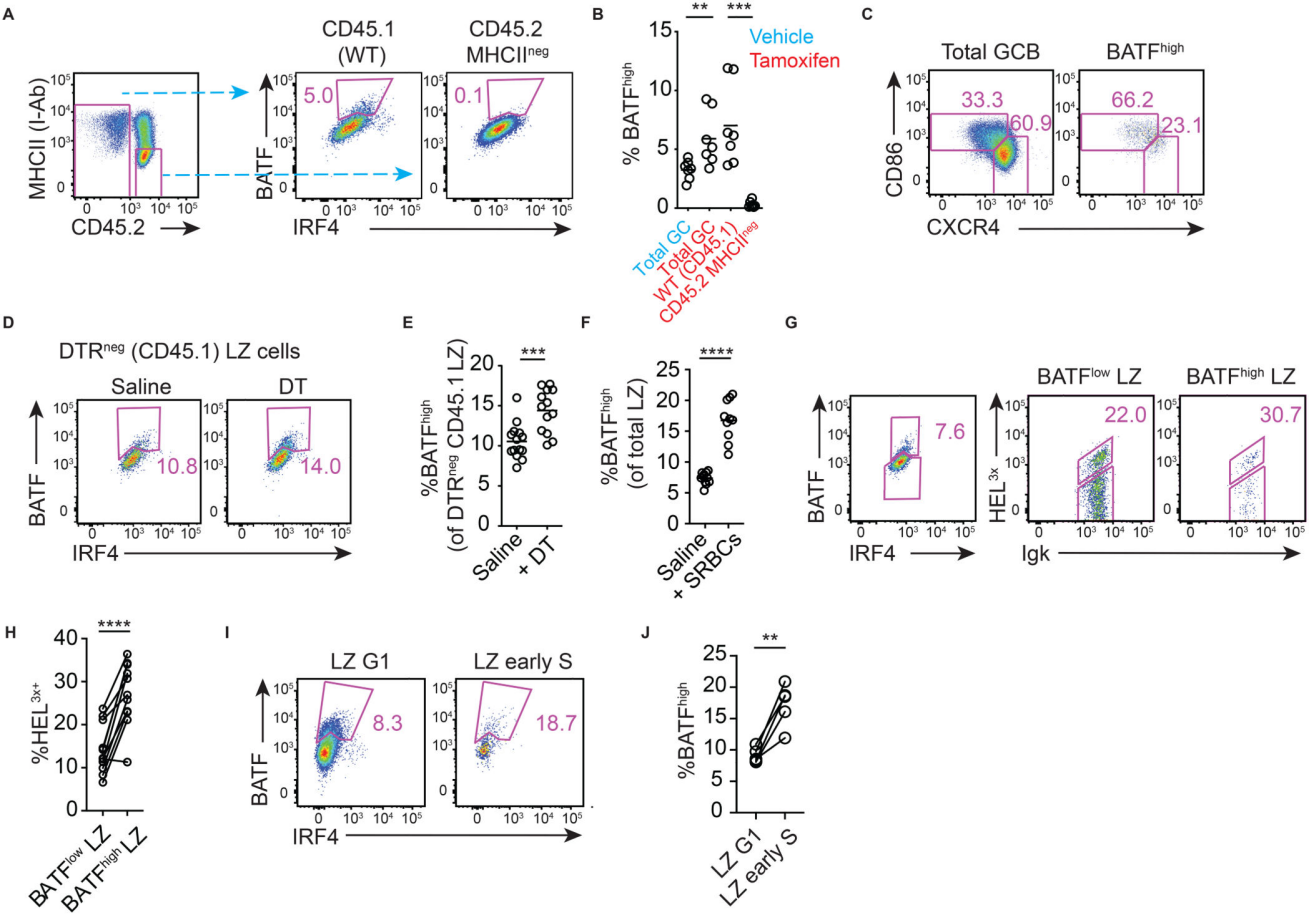
BCR affinity, cell-cell competition and intrinsic antigen acquiring capacity determine T cell-mediated refuelling in LZs. (A) BATF induction by WT and MHCII-deleted GC (IgD^low^ CD95^+^ GL7^+^) cells in WT: CreERT2^+^ MHCII^fl/fl^ on day 8 post-SRBC immunisation, 24hrs after tamoxifen treatment, and (B) enumeration. (C) LZ and DZ gating applied to total GC B cell and BATF^high^ populations. (D, E) Frequencies of WT (DTR^neg^) LZ cells that induced BATF on day 8 post-SRBC immunisation, 15hrs after partial GC ablation to reduce competition (WT:AID-DTR 20:80 BM chimeras). (F) SRBC immunised WT mice received second SRBC injections (or saline) on day 7, and frequencies of BATF^high^ LZ cells were assessed on day 8. (G, H) Analysis of splenic SW_HEL_ GC B cells (CD45.1 IgD^low^ GL7^+^) responding to HEL3x-SRBC/LPS (or HEL^WT^-SRBCs, staining control) immunization, on day 8. Frequencies of HEL^3X^-bright cells within BATF^low^ and BATF^high^ LZ populations are shown. (I-J) Frequencies of BATF^high^ cells within G1 (Edu^-^ BrdU^-^) and early S (EdU^-^ BrdU^+^) LZ populations were determined in SRBC immunised WT mice on day 7. Mice received EdU and BrdU treatments 100 mins and 40 mins before analysis. A, C, D, G, I are representative plots. Pooled results from 2 (B), 3 (E), 3(F), 3(H), 2(J) independent experiments. Lines in (H, J) join points from individual mice. Analysis, (B, E, F) unpaired and, (H, J) paired, two-tailed Student’s t test. **p < 0.01, ***p < 0.001, ****p<0.0001.

A method was required for identifying recently helped (or refuelled) cells directly *ex vivo*. Guided by published findings (37), we determined that BATF is induced in GC B cells in an MHCII and CD40L dependent manner (Fig. 6A-B, S4A-B), providing a convenient measure. These findings also further validated that experimental MHCII-deletion prevents functional T-B interactions as early as 24hrs after tamoxifen treatment. High BATF expression in the GC was restricted mostly to LZ cells, consistent with

this being the site of positive selection (Fig. 6C) (37). In line with an earlier report (38), optimal c-Myc protein expression (number of positive cells and expression level per cell) also required MHCII expression (Fig. S4C-E), while residual MHCII-independent c-Myc expression (but not cyclic re-entry initiation, Fig. S4F) was highly sensitive to chemical Btk-inhibition (ibrutinib treatment), suggesting likely BCR signaling involvement. Interestingly, Ibrutinib partially mimicked genetic Syk deletion (38, 39), in reducing LZ sizes (Fig. S4G). Small populations of BATF^low^ IRF4^high^ cells were also evident in MHCII^+^ but not MHCII-deleted populations (Fig. 6A), however we believe these represent mixes of pre-plasma cells and contaminating plasma cells that may have delayed MHCII turnover rates due to lower *March1* expression (Immgen.org) (26, 29).

We initially planned to test the impact of competition by deleting MHCII from competitor cells, however tamoxifen treatment alone caused BATF increases (example evident in Fig. 6B, similar findings were reported for c-Myc (17)). We therefore returned to the partial GC ablation strategy (as in Fig. 4). 15hrs post-DT treatment, the frequency of BATF^high^ CD45.1 WT LZ cells was increased by ~1/3 (mean 14.4% vs 10.6%) when competition was relieved by ~70% (Fig. 6D-E, S4H-I), supporting the concept that competition between GC B cells restricts T cell access, even at physiological pMHCII presentation levels. The relatively modest changes in BATF^high^ cell frequencies (~36%) compared to the level of competition relief provided (~70% reduction) led us to test whether similar changes would occur when smaller fractions (~50%) of the competitors were removed, but again increases in BATF^high^ cell frequencies were evident (Fig. S4J, K). Despite this, however, our findings also argue for a more complex situation in which the local availability of antigen, and by inference the intrinsic ability of B cells acquire it, also play major roles in determining amounts of refuelling received because simply injecting extra antigen one day before analysis caused striking increases (a doubling) in the frequencies of BATF^high^ LZ cells (Fig. 6F, Fig. S4L) (without increasing cyclic re-entry rates, Fig. S4M).

Finally, we confirmed that high affinity GC B cells were more likely to succeed in receiving T cell support, because BATF induction was associated with BCR affinity in SW_HEL_ LZ populations (Fig. 6G, H). Together, these results support the concept that T cells, while not acutely required for cyclic re-entry initiation, instruct various critical molecular pathways in GC B cells in an affinity-dependent manner that reflects how efficiently a B cell gathers antigen as well as how it competes with cohabiting B cells. Consistent with this, early S phase LZ populations were enriched with BATF^high^ cells but also contained others (a majority) in which BATF had not been detectably induced (Fig. 6I, J).

### Lower affinity GC B cells spend longer in light zones before initiating cyclic re-entry

We noted that, where cyclic re-entry initiation was tracked in Fucci2 mice, LZ cells entered S phase from a range of mCherry levels spanning ~½-log (e.g., Fig. 1C), suggesting that cells may spend differing amounts of time “resting” in LZs. Using the same EdU pulse-chase approach described earlier (Fig. S1), we validated this interpretation because mCherry intensities in early S phase LZ populations faithfully reflected time post-mitosis (Fig. S5A, B). These experiments also provided quantitative information about how long cells remain in G1 prior to initiating cyclic re-entry, with the “earliest” LZ cells entering S phase within just ~10hrs of last replicating their DNA, and >60% of cells doing so within 15hrs (Fig. S5C). Given that GC B cells mostly downregulate CXCR4 for LZ entry ~8hrs after completing their last DZ S phase (6, 9, 10), these results suggest that the “fastest” GC B cells may initiate cyclic re-entry only a small number of hours after that.

**Figure 7:**
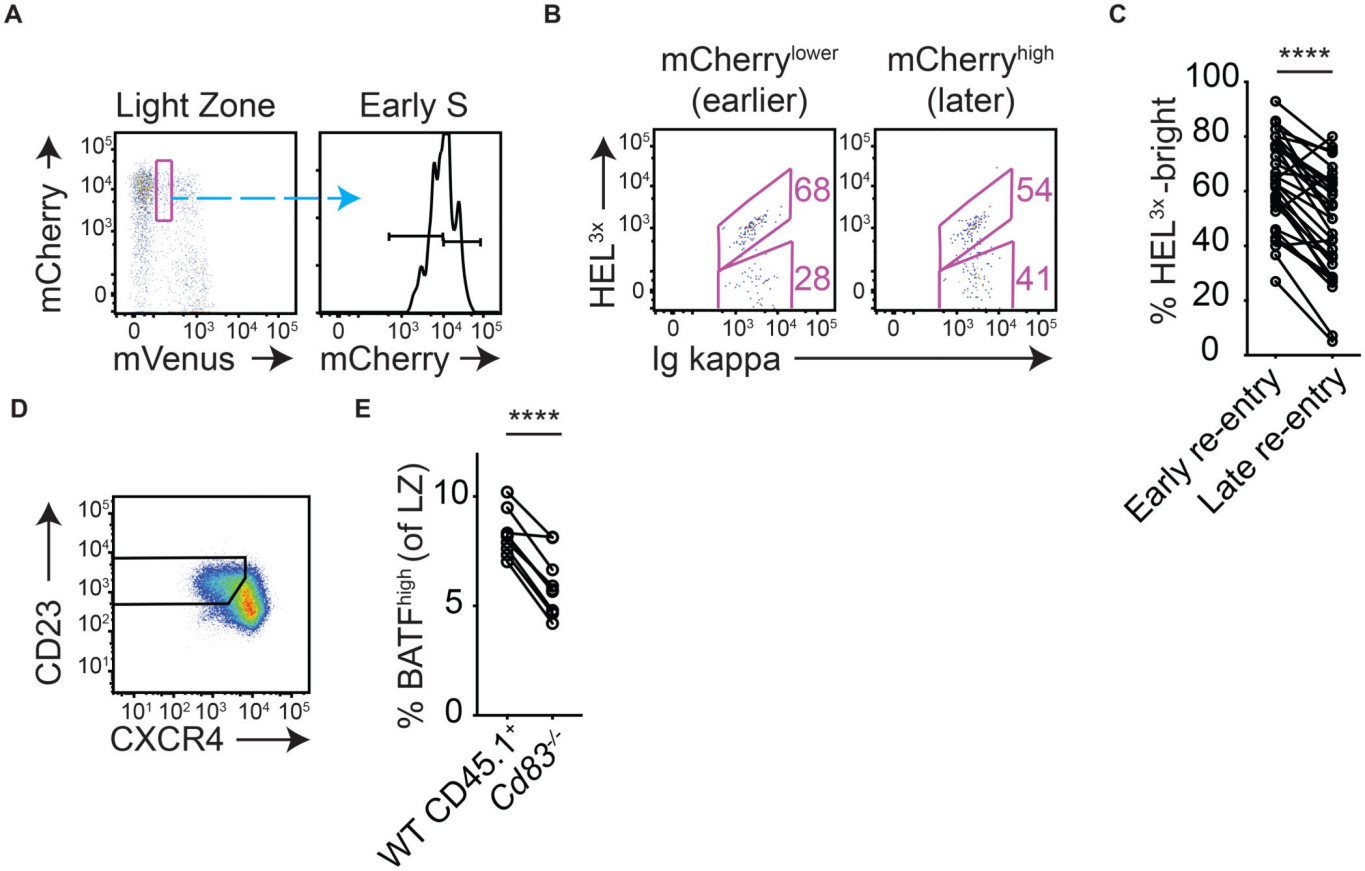
Lower affinity cells spend more time resting in LZs before initiating cyclic re-entry. (A, B) Analysis of splenic SW_HEL_ Fucci2 GC B cells responding to HEL3x-SRBC/LPS (or HEL^WT^-SRBCs, staining control) immunization, on day 8. Very early S phase cells were subdivided into lower (“early”) and higher (“later”) ~50 percentiles based on their mCherry levels (B) Frequencies of HEL_3x_-bright cells were determined for each of the populations. (C) Summary of pooled results from multiple experiments. (D, E) Day 8 post-SRBC immunisation in *WT:Cd83^-/-^* mixed BM chimeric mice. (D) LZ populations were identified and, (E) frequencies of BATF^high^ cells were determined. A, B, D are representative FACS plots, lines in C, E join populations from single mice. C, E is pooled from 6 and 2 experiments, each containing 3-6 mice. Analysis, paired two-tailed Student’s t test. ****p < 0.0001.

To ask whether BCR affinity is associated with LZ rest periods, we again utilized the Fucci2 SW_HEL_/HEL^3x^ staining approach. LZ cells at the earliest detectable S phase stages were gated using very stringent mVenus^low^ gates (Fig. 7A) to catch them before mCherry degradation occurs. These populations were further subdivided into lower and higher mCherry fractions, and the frequencies of HEL^3x^-bright cells were determined (Fig. 7B, C). Strikingly, high affinity cells were enriched within the mCherry-lower (“earlier”)

S phase population (Fig. 7B, C). A graded affinity-dependent effect was apparent when late G1 and early S phase populations were further divided into four subsets (Fig. S5D-F). Consequently, the mCherry-highest (latest) “resting” G1 populations were highly enriched for low affinity cells. These subsets are expected to be enriched for cells that are destined for memory differentiation (31–33, 40). LZs also contain small atypical subsets of cells that seemingly complete mitosis in that zone and remain there for some period (early G1 cells, Fig. 7A, 1C, S1C) (9, 41); whether such cells later rejoin DZs, or instead represent a differentiating subset such as proliferating pre-plasma cells (26) (41) (42), is not clear. A comprehensive overview of associations between BCR affinity/cell cycle stages is provided in Fig. S6A, B.

The described observations suggested that individual LZ cells may be afforded different periods for accumulating antigen and refuelling before initiating cyclic re-entry. It was particularly striking that low affinity cells spend longer resting in LZs because this could contribute to the generation and retention of clonal diversity by providing them more time to accumulate antigen and interact with T cells (albeit with the sacrifice of slight delays in proliferative progression). While tools do not exist for controlling LZ dwell times to test how it impacts T cell-mediated refuelling, “later” cells will benefit most from mechanisms preventing pMHCII turnover at that stage and therefore we believed it important to know whether such pathways directly influence refuelling. CD83 expression in LZ cells stabilizes pMHCII complexes by antagonizing March1-mediated ubiquitination and degradation (29, 43, 44). LZ cells from *WT/Cd83*^-/-^ BM chimeras were gated using a CXCR4^low^ CD23+ strategy (Fig. 7D) (4), and the frequencies of BATF^high^ cells were determined. Consistent with the proposed hypothesis, frequencies of BATF^high^ cells were higher among populations that were able to use CD83 for stabilizing MHCII complexes (Fig. 7E). Therefore, spending more time in the LZ state may promote the refuelling of lower affinity cells.

## Discussion

Here, we revisited the central tenets that clonal selection in GCs involves B cells needing to compete to initiate cyclic re-entry each time they enter LZs, and that the provision of T cell help is the essential and limiting factor for this. We had expected that LZ cells prevented from forming cognate T-B interactions would fail to leave their resting late G1 state, however neither the depletion of most T_fh_ cells nor the deletion of MHCII prevented cell cycle entry. This led us to test whether this cyclic re-entry initiation step is restricted by competition for other T cell-independent selection factors, but we also failed to find evidence for this. In contrast, the same perturbations dramatically changed the likelihood of cells upregulating BATF. BATF is an important DZ sustaining transcription factor (37), and a robust molecular marker of having received T cell help. As such, we propose a revised model for GC B cell selection.

The focus of our study was positive selection in LZs, however we consider our revised model from the point of SHM in DZs. The first challenge which newly mutated GC B cells face is to try and re-express a functional surface BCR, with cells failing this step mostly undergoing apoptosis before DZ egress (10, 12). The molecular mechanisms responsible for this “negative” selection event are not yet known, however our SW_HEL_/Fucci2 tracking experiments further support that BCR affinity is not a determinant, supporting a likely role for tonic signaling (45). The rapid deletion in DZs of these unredeemable clones may be important for allowing LZ positive selection events to be permissive, because it negates the need for an all-or-nothing clearance mechanism. Cells passing this step downregulate CXCR4 and enter LZs in a resting late G1 state, coincident with gene and protein expression changes that reduce SHM, de-repress key signaling pathways and re-tune their cellular machinery for positive selection (4, 29). LZ cells migrate within the immune complex laden FDC network and are provided the opportunity to gather intact antigen and compete to establish a series of short cognate interactions with T_fh_ cells (9, 46).

**Figure 8:**
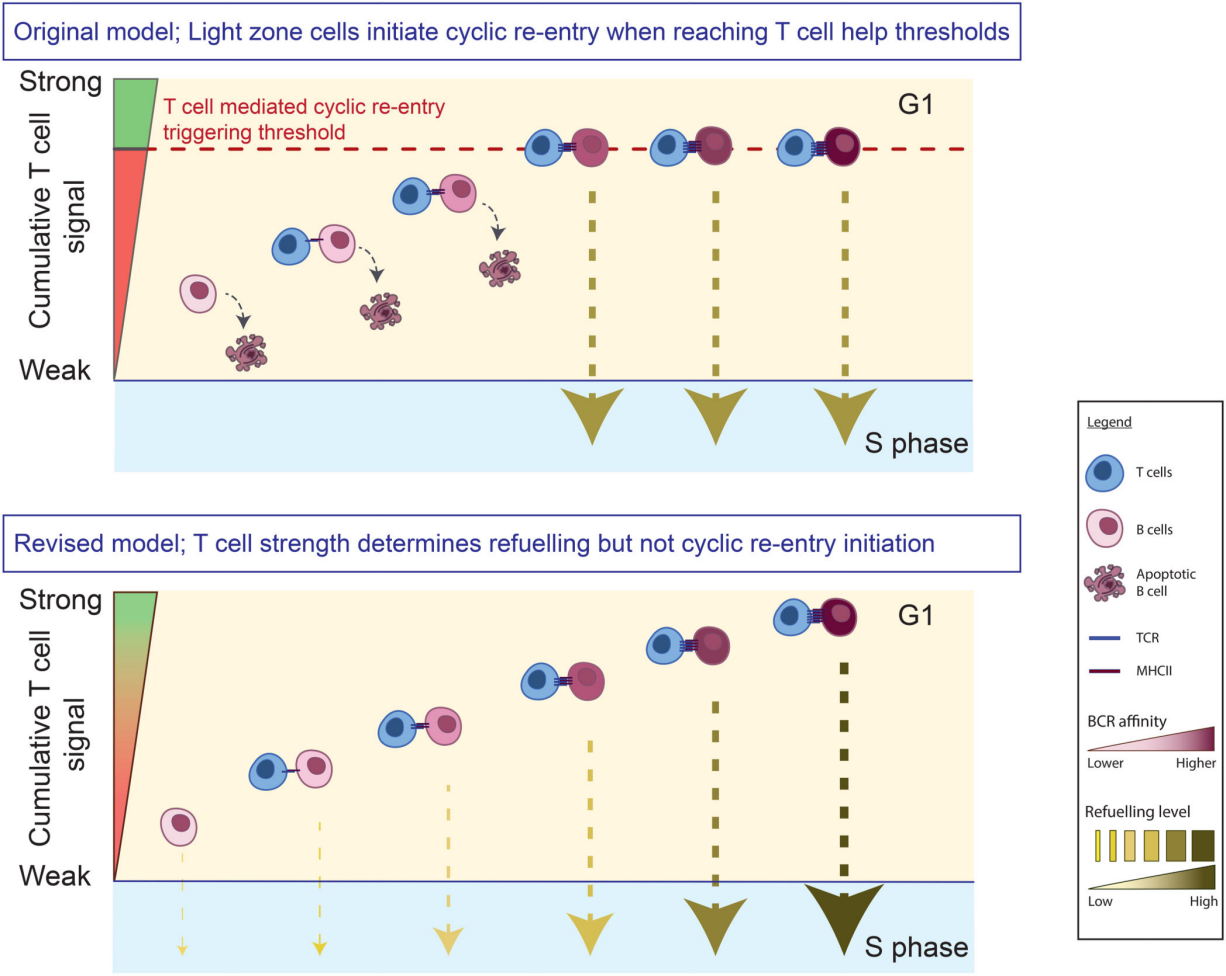
Alternative models for how T cells support antibody affinity maturation. GC B cells enter LZs in G1, where they attempt to establish transient interactions with T_fh_ cells. In one scenario (top), LZ cells reaching a certain threshold of T cell help (dashed line) are triggered to initiate cyclic re-entry. Most cells fail this step and subsequently apoptose, which excludes all but the fittest cells. T cell help strength also determines the subsequent division capacity of cells, however because cyclic re-entry is triggered at a certain help threshold, most cells may undergo this event having received similar doses. Alternatively (bottom), cyclic re-entry initiation is regulated independently of strength of T cell help received (our proposed model). As a result, both lower and higher affinity cells can initiate cyclic re-entry, with cells doing so having been refuelled to differing degrees (which determines division capacity, indicated by arrow weight). Note, our experimental measure of cyclic re-entry initiation was restarting of cell cycle (S phase entry), therefore whether cells can complete cyclic re-entry (i.e., return to DZs) without adequate T cell help remains to be tested.

GC selection must preferentially expand high affinity B cells at the same time as nurturing polyclonal responses to both immunodominant and subdominant epitopes (2, 3, 47). An important recent discovery was that T_fh_ cells select LZ cells to proliferate to differing degrees based on the cumulative strength of the help provided (15, 17, 18). This non-binary selection mode may contribute to explaining how clonal diversity is supported, however it also posed a conceptual problem in the context of traditional selection models. If cyclic re-entry initiation is itself triggered by LZ cells reaching certain T cell help signaling thresholds, one might assume that most cells will return to DZs with very similar division capacities (Fig. 8, top). Our new findings resolve this conundrum because we find that cyclic re-entry initiation is regulated independently of the inputs known to determine cellular division capacity (Fig. 8, bottom). Importantly, because this selection mode is non-binary, we also no longer need to assume that all but the very highest affinity (or best competing) cells are purged from responses each time they enter LZs. Our findings clearly showed that cell-cell competition contributes to determining how much help cells receive, even at physiological pMHCII levels, however antigen availability (and by inference BCR affinity) was also limiting. As such, LZ cells acquiring atypical amounts of antigen, e.g., due to affinity enhancing mutations or stochastic encounters with large antigen fragments (2), may be released from their normal constraints and expanded quickly, which we speculate might contribute to the seeding of clonal bursts (22). Throughout this study we have referred to the receiving of T cell help as refuelling (25); this broad term was favored to encompass pathways that control cell division number (e.g. Cyclin D3, c-Myc), as well as others that do not directly regulate cell cycle progression but are required for anabolic growth and sustenance within the DZ (e.g., mTORC1, BATF) (37, 48, 49). It also implies an ability to top up from a partially fuelled state, something we speculate may be possible. Consistent with the proposed model, cells initiated cyclic re-entry refuelled to different degrees (i.e., early S phase LZ cells, only some of which had high BATF levels) even in an unperturbed settings. Prior studies established that c-Myc levels can also vary at this stage (17, 48). Recent mathematical modelling efforts support the importance of separating cyclic re-entry and pro-proliferation regulation (50).

A key unanswered question that arises is what inputs or pathways, if not those provided directly by T cells, determine if and when LZ cells will re-enter S phase? We imagine only two possible mechanisms; either cyclic re-entry initiation occurs when non-competitive, BCR-dependent signaling thresholds are reached, or it is regulated by cell-intrinsic pathways set into play at an earlier time, a form of “inertia”. In support of the former, cyclin D3 is required specifically for GC B cells to pass the G1/S checkpoint in DZs but not LZs (18, 19), arguing that cell cycle initiation can be discernably different in the two zones. Interestingly, the early stages of plasma cell differentiation can sometimes be triggered by BCR-dependent, but T-cell independent, pathways (51), with T cells playing a more qualitative role later on (26). If a similar process determines cyclic re-entry initiation, we must then question why don’t more (or even all) LZ cells re-enter S phase (or trigger plasma cell differentiation) when antigen availability is augmented? One explanation might be that individual LZ cells differ in how they respond to external cues, possibly as a result of differences in signaling circuit connections or epigenetics (26, 52). Our efforts to block BCR signaling *in vivo* using pharmacological inhibition, while sufficient to reduce c-Myc levels to undetectable levels when combined with MHCII deletion, did not prevent cyclic re-entry initiation. However, it is unlikely that any such chemical block is absolute, therefore definitively answering this question will require new genetic tools. Importantly, if we ultimately find that cyclic re-entry initiation occurs independently of antigen-specific BCR inputs, i.e., the inertia model proves correct, this would mean it is not always an immediate consequence of selection events. Until then, we remain agnostic in regards to these possibilities.

Our study has clear limitations, most notably that we followed a critical but single measure of cyclic re-entry, S phase entry from a resting LZ state. As such, that unhelped LZ cells ultimately return to DZs remains to be proven. For this to occur, GC B cells must activate transcriptional pathways that control the DZ program, most notably FOXO1 (37, 53, 54). Whether this itself depends on specific external activating cues, or instead happens by default when negative regulation by PI3K ceases, is not clear. Other molecular players such as BATF and mTORC1 are also needed for DZ sustenance (25, 37); regarding these, we speculate that unhelped cells may temporarily persist on “reserves” from earlier selection events. For example, most LZ cells have higher than basal BATF levels (compared to *Batf^-/-^* controls) (37), and they also maintain DZ-like pS6 levels (that are higher than rapamycin treated controls) even when CD40L signaling is blocked (25). Interestingly, mTORC1 hyperactivation has been shown to sometimes reduce T cell dependence (55). It may also be relevant that GC B cells can sometimes persist for prolonged periods having experienced a only single antigenic encounter (56). However, while we are certainly interested in the fate of unhelped cells, the MHCII-deletion approach described herein in an experimental extreme; how often “normal” GC B cells find themselves completely unhelped (rather than poorly helped) is not clear. Therefore, the more important point to emphasize is that our findings do not support the concept that initiating cyclic re-entry depends on reaching stringent T cell help thresholds, and that this is important because it may help explain how cells undergo this process having been refuelled to varying degrees (17).

An important unexplained finding from our study is that fewer LZ cells initiate cyclic re-entry at late time points after MHCII deletion, despite the process seemingly not being acutely T cell dependent. A trivial explanation would be that T cell help is required for cyclic re-entry initiation, but that “MHCII-deleted” LZ cells lost their surface MHCII proteins only after cyclic re-entry was triggered. However, multiple lines of evidence all but exclude this possibility. Firstly, LZ cells are very unlikely to be afforded sufficient time for this to frequently occur; DZ cells typically downregulate CXCR4 and move into LZs ~8hrs after completing mitosis (6, 9, 10), yet more than half of all LZ cells initiated cyclic re-entry within 12hrs of completing their last division. As such, cells generally spend no more than a few hours in LZs before entering back into cell cycle (and MHCII turnover is slow at that stage (29)). Other arguments include that unhelped cyclic re-entry initiation was evident even at 6 days post-tamoxifen treatment, when MHCII deletion was seemingly complete, and that neither increasing T-B ratios nor providing more antigen, both of which increased T cell mediated refuelling, enhanced cyclic re-entry initiation rates. As such, the issue of why cyclic re-entry initiation rates decrease at later time points remains unresolved. Possible (non-mutually exclusive) explanations include that selection cues received during earlier LZ cycles may influence subsequent steps, or that (while not required) the provision of T cell help can provide a second route of cyclic re-entry initiation for cells that would otherwise remain in G1 (e.g., cells without metabolic reserves). This latter possibility could represent a “have another go” route for response retention, and it might represent an augmented route to clonal expansion for certain higher affinity cells.

A further finding reported herein is that individual LZ cells rest in G1 for different time periods before restarting S phase, with the rest period duration being linked to BCR affinity. On average, high affinity cells initiated cyclic re-entry earlier. We speculate that this may make it easier for lower affinity cells to be refuelled, because they will have more time to collect antigen and engage with T cells. At face value, these observations are at odds with prior findings that enhancing antigen presentation by GC B cells causes temporary pausing in LZs (4), however we suspect that this pausing behaviour may reflect what happens periodically for cells receiving stronger than typical doses of help, possibly prior to them going on to establish clonal dominance (2, 22). We also cannot exclude that strong T cell help may sometimes cause GC B cells to remain in LZs after completing mitosis (41), and that these cells are then among those re-entering S phase at “earlier” time points in the next cell cycle.

In summary, by directly identifying GC B cells initiating cyclic re-entry and testing the minimal requirements for this to happen, we unexpectedly found that this process can occur independently of acutely received T cell-derived selection inputs and appears not to be directly influenced by competition. Instead, our findings support a model in which affinity improvements are favoured by T cells preferentially providing stronger refuelling cues to B cells that express better antibodies. This less restrictive form of selection may act in concert with other phenomenon such as antibody feedback dependent epitope masking (57), bystander cytokine signalling (58), and stochasticity at the point of antigen encounter (2), to enable moderate affinity cells to persist in GCs for longer and ultimately help support the complex antibody maturation pathways, such as those required for HIV broadly neutralising Abs development (20).

## Materials and methods

### Study Design

The aim of this study was to investigate if competion between GC B cells for T cell help are key and limiting factors determining whether or not GC B cells initiate cyclic re-entry when they enter LZs. The fractions of LZ cells in early S phase were assessed after T cell ablation in Fucci2 T cell deficient mice that had received ablatable T cells by adoptive transfer. Similar assessments were made after MHCII deletion from GC B cells, as well as when competing cells were experimentally ablated (Rosa26-CreERT2^+^ MHCII^fl/fl^/B6.SJLCD45.1 and Aicda^wt/cre^ R26-loxp-stp-loxp-DTR/ B6.SJLCD45.1 mixed bone marrow chimeric mice, respectively). The correlation between BCR affinity and cyclic re-entry initiation and T cell-mediated refuelling was subsequently tested. The competition between B cells to receive T cell mediated refuelling was confirmed by partial B cell ablation experiments. Sample sizes and numbers of replicates for each experiment are indicated in the figure legends. Sample sizes were determined based on power calculations, guided by earlier similar studies from our lab. Only T cell ablation experiments required data exclusion, whose criteria was detailed in the Immunisations, treatments and adoptive transfers section. Outliers were not excluded.

### Mice

SW_HEL_ (59), Rosa26-CreERT2^+^ (28), I-Ab (*H2-Ab1*)^fl/fl^ (27), Aicda^wt/cre^ (60), R26-loxp-stp-loxp-DTR (61), CD79a-CreERT2 (62) (63), OT-II (64), TCR-b^-/-^ and b/d^-/-^ (65) (66) and R26p-Fucci2 (Riken accession CDB0203T, http://www.clst.riken.jp/arg/reporter_mice.html) (24), Nur77-GFP (35), CD4-Cre (67), mice have been described previously. C57BL/6 and B6SJL.CD45.1 mice were purchased from the University of Oxford core breeding facility. All mice were >6 weeks of age at time of experimentation, and mixes of male and female mice were used. We occasionally noted that Rosa26-CreERT2^+^ x MHCII^fl/fl^ mice to have universally low surface MHCII levels without tamoxifen treatment, possibly indicating germline Cre activity, therefore I-Ab protein levels were screened before use and careful breeding strategies were employed.

All mice were bred and maintained in specific pathogen–free facilities at Biomedical Sciences Facility at the University of Oxford. All experiments were performed under the authorization by a project license granted by the UK Home Office and were also approved by the Institutional Animal Ethics Committee Review Board at the University of Oxford.

### Mixed bone marrow chimeras

A mixture of C57BL/6 and B6.SJLCD45.1 mice >8 weeks of both sexes were used as recipients for mixed BM chimeric mice. Recipient mice were lethally irradiated twice at 4.5 Gy dose for 300 s, separated by a ~3 hour rest. Mice were then injected i.v. with mixes of 80% Rosa26-CreERT2^+^ MHCII^fl/fl^/20% B6.SJLCD45.1 (target frequencies), 60% Rosa26-CreERT2^+^ MHCII^fl/fl^ Fucci2/40% Fucci2 B6.SJLCD45.1, 80% Aicda^wt/cre^ R26-loxp-stp-loxp-DTR/20% B6.SJLCD45.1, 55% Aicda^wt/cre^ R26-loxp-stp-loxp-DTR/45% B6.SJLCD45.1, 80% Aicda^wt/cre^ R26-loxp-stp-loxp-DTR/20% R26p-Fucci2 or 80% Aicda^wt/cre^ R26-loxp-stp-loxp-DTR/20% Nur77-GFP CD45.1 BM cells, respectively with donor mice being sex matched to each other. Recipient mice received antibiotics (0.16mg/mL, Enrofloxacin (Baytril), Bayer Corporation) in drinking water for four weeks following irradiation and were rested for >8 weeks before experiments.

### Immunisations, treatments and adoptive transfers

For HEL-specific GC responses, 1x10^5^ SW_HEL_, MHCII^fl/fl^ x CD79a-CreERT2 SW_HEL_ or SW_HEL_ x R26p-Fucci2 cells were transferred into C57BL/6 or B6SJLCD45.1 hosts by i.v. injection. Mice also received equal numbers of OT-II cells in experiments where HEL-OVA was used. Mice were immunised with HEL^3x^ (or HEL^WT^) conjugated to SRBCs (HEL-SRBCs, including 10ug/ml LPS) (36) or 50ug HEL-OVA chemically conjugated as previously described (9) in sigma adjuvant system (Sigma Aldrich, S6322) 1-3 days later (SRBCs in Alsevier’s solution used). Mice receiving SW_HEL_ B cells and SRBC immunisation did not establish CD45.1 GC B cells, confirming that the low affinity cells detected by FACS were specific for HEL^3x^ and not other SRBC epitopes. The HEL^3x^ gene (36), with two C’-6xHis-tags following the signal sequence, was cloned into a pHR-ires-GFP lentiviral vector, CHO cells were transduced, and cells with high expression (GFP^bright^) FACS sorted and expanded. HEL^3x^ protein was harvested from the supernatant and purified over a NiNTA column. HEL^WT^ was purchased from Sigma Aldrich, cat no.62971.

For T cell transfer experiments, splenocytes from CD4-Cre^+^ DTR^+^ donor mice were were transferred into sex-matched recipients (~1 donor per 3 recipients), which were subsequently immunised with defibrinated SRBCs (90ul/mouse, diluted in saline to 300ul, Fisher Scientific, i.p.) supplemented with LPS (Sigma Aldrich, L6529) (10 ug/ml). Animals were excluded from analysis when T cell ablation was not effective (CD4^+^ TCRb^+^ T cells were >0.4% of splenocytes).

For polyclonal SRBC responses, mice were immunised with ~2-3 x 10^8^ defibrinated SRBCs by i.p. injections (90ul/mouse, diluted in saline to 300ul, Fisher Scientific, i.p.). For NP-KLH (Biosearch Technologies, N-5060-5) immunisations, antigen was absorbed in Anhydrogel alum (Invivogen, vac-alu-250) and injected i.p‥ For BrdU (Sigma Aldrich, B5002) and EdU (Biosynth Carbosynth, NE08701) treatments, mice received single i.p. injections of 2mg BrdU or 1 mg EdU in saline at the indicated periods before tissue harvest. For DT treatment, mice received single doses of 0.75 μg DT (CalbioChem) in saline (or vehicle alone) at indicated time points by i.p. injection. Tamoxifen (2mg/mouse, Sigma) was dissolved in 10% ethanol/90% corn oil, sonicated and delivered by i.p. injection. For anti-CD40L antibody treatments, mice received 0.5mg antibody (MR1 clone, Biolegend) in saline by i.p. injections 24hrs before tissue harvest. Ibrutinib (Selleckchem.com) treatments were provided through 2 i.p. injections of 0.25mg/mouse Ibrutinib, separated by 3 hrs. Ibrutinib was dissolved in Captex 355 (Abitec) (1.56mg/ml) and further diluted in PBS prior to use.

### Flow cytometry and FACs sorting

Spleens were passed through 40μm cells strainers to generate single cell suspensions and stained for with the relevant antibodies. For BATF and IRF4 staining, cells were fixed (30 minutes) and permeabilized with the Foxp3/Transcription Factor Staining buffer (ThermoFisher Scientific) prior to intracellular staining. For Y-ae staining, splenocytes were cultured *ex vivo* in DMEM/10% FBS/Hepes/Pen/Step with HEL-EaGFP or HEL-BSA at 3ug/mL for 2 hrs at 37°C prior to antibody staining. For BrdU stains, cells were fixed/permeabilized with BD BrdU flow kit following manufacturer’s instructions, and stained using the 3D4 antibody. EdU staining was performed on samples fixed for 30 mins with BD Cytofix/Cytoperm, following manufacturer’s instructions (Click-iT™ Plus kit, ThermoFisher Scientific). All permeabilization steps included a short vortex pulse and were performed with an overnight incubation at 4°C. Data acquisition was carried out using BD LSR II, LSR Fortessa, LSR Fortessa X20, FACSymphony cytometers. FACs sorting was performed using the FACs Aria II SORP or Fusion II machines with 85um or 100um nozzles. Analysis was performed with Flowjo (Treestar Inc.).

### Antibodies

The following monoclonal antibodies against mouse B220 (RA3-6B2), IgD (11-26c.2a), GL7 (GL7), CD3e (145-2C11), CD86 (GL1), CD83 (Michel-19), IRF4 (IRF4.3E4), I-Ab (AF6-120.1), IgKappa (RMK-45), CD45.1 (A20), CD45.2 (104), CD4 (GK1.5), TCRb (H57-597), CD44 (IM7), CD62L (MEL-14), PD1 (29F.1A12) were purchased from Biolegend. The mAbs against mouse CXCR5 (2G8), CD95 (Jo2) were purchased from BD Biosciences. Anti-mouse BATF (D7C5) was obtained from Cell Signaling Technology. Anti-mouse CXCR4 (2B11), Donkey Anti-Rabbit IgG AF647, Anti-Mouse Ea52-68 peptide (Y-ae), Streptavidin Qdot605 (Q10101MP), were purchased from Thermo Fisher Scientific.

### Immunostaining

Spleen fragments were snap frozen in O.C.T. mounting medium (Tissue-Tek). 7 or 10 uM cryostat sections were cut and fixed with ice-cold acetone (10 mins) prior to drying for >1hr. For immunohistochemistry (IHC), slides were subsequently rehydrated in TBS with 0.1% BSA and stained for >3hrs with the relevant antibodies in TBS/blocking serums. Secondary stains were performed for >1hr. Enzyme conjugates were developed using Sigma Fast DAB and Fast Blue substrates prior to mounting with Aqua-mount. For immunofluorescence (IF), slides were rehydrated in PBS/0.1% BSA and stained for >3hrs with antibodies in PBS/blocking serums, washed and stained with secondary reagents (>3hrs). IHC images were acquired using the Echo Revolve microscope and IF images were acquired with 10x Axioscan (Zeiss).

### Statistical analysis

Shapiro-Wilk normality testing was first used to determine the normality of each experiment dataset then. statistical significance was determined by unpaired, two-tailed Student’s t test, Mann-Whitney test, Kruskal-Wallis one-way ANOVA with multiple comparisons or Wilcoxon paired signed rank test as appropriate. The statistical method used is indicated in the respective figure legends. P < 0.05 was considered as statistically significant. Prism 8 software (Graphpad) was used to calculate all statistical analyses.

## Supplementary Material

Supplementary Material

## Data Availability

All data needed to evaluate the conclusions in the paper are present in the paper or the Supplementary Materials.

## One sentence summary

Germinal center B cells compete to be refuelled by T cells but not for cyclic reentry initiation during affinity maturation.

## References

[1] Victora GD, Nussenzweig MC (2012). Germinal centers. Annu Rev Immunol.

[2] Mesin L, Ersching J, Victora GD (2016). Germinal Center B Cell Dynamics. Immunity.

[3] Bannard O, Cyster JG (2017). Germinal centers: programmed for affinity maturation and antibody diversification. Curr Opin Immunol.

[4] Victora GD, Schwickert TA, Fooksman DR, Kamphorst AO, Meyer-Hermann M, Dustin ML, Nussenzweig MC (2010). Germinal center dynamics revealed by multiphoton microscopy with a photoactivatable fluorescent reporter. Cell.

[5] Bannard O, Horton RM, Allen CD, An J, Nagasawa T, Cyster JG (2013). Germinal center centroblasts transition to a centrocyte phenotype according to a timed program and depend on the dark zone for effective selection. Immunity.

[6] Liu YJ, Zhang J, Lane PJ, Chan EY, MacLennan IC (1991). Sites of specific B cell activation in primary and secondary responses to T cell-dependent and T cell-independent antigens. Eur J Immunol.

[7] Kepler TB, Perelson AS (1993). Cyclic re-entry of germinal center B cells and the efficiency of affinity maturation. Immunol Today.

[8] MacLennan IC (1994). Germinal centers. Annu Rev Immunol.

[9] Allen CD, Okada T, Tang HL, Cyster JG (2007). Imaging of germinal center selection events during affinity maturation. Science.

[10] Stewart I, Radtke D, Phillips B, McGowan SJ, Bannard O (2018). Germinal Center B Cells Replace Their Antigen Receptors in Dark Zones and Fail Light Zone Entry when Immunoglobulin Gene Mutations are Damaging. Immunity.

[11] Meyer-Hermann M, Mohr E, Pelletier N, Zhang Y, Victora GD, Toellner KM (2012). A theory of germinal center B cell selection, division, and exit. Cell Rep.

[12] Mayer CT, Gazumyan A, Kara EE, Gitlin AD, Golijanin J, Viant C, Pai J, Oliveira TY, Wang Q, Escolano A, Medina-Ramirez M (2017). The microanatomic segregation of selection by apoptosis in the germinal center. Science.

[13] Allen CD, Okada T, Cyster JG (2007). Germinal-center organization and cellular dynamics. Immunity.

[14] Batista FD, Neuberger MS (2000). B cells extract and present immobilized antigen: implications for affinity discrimination. Embo j.

[15] Gitlin AD, Shulman Z, Nussenzweig MC (2014). Clonal selection in the germinal centre by regulated proliferation and hypermutation. Nature.

[16] Gitlin AD, Mayer CT, Oliveira TY, Shulman Z, Jones MJ, Koren A, Nussenzweig MC (2015). HUMORAL IMMUNITY. T cell help controls the speed of the cell cycle in germinal center B cells. Science.

[17] Finkin S, Hartweger H, Oliveira TY, Kara EE, Nussenzweig MC (2019). Protein Amounts of the MYC Transcription Factor Determine Germinal Center B Cell Division Capacity. Immunity.

[18] Pae J, Ersching J, Castro TBR, Schips M, Mesin L, Allon SJ, Ordovas-Montanes J, Mlynarczyk C, Melnick A, Efeyan A, Shalek AK (2021). Cyclin D3 drives inertial cell cycling in dark zone germinal center B cells. J Exp Med.

[19] Ramezani-Rad P, Chen C, Zhu Z, Rickert RC (2020). Cyclin D3 Governs Clonal Expansion of Dark Zone Germinal Center B Cells. Cell Rep.

[20] West AP, Scharf L, Scheid JF, Klein F, Bjorkman PJ, Nussenzweig MC (2014). Structural insights on the role of antibodies in HIV-1 vaccine and therapy. Cell.

[21] Zhou D, Dejnirattisai W, Supasa P, Liu C, Mentzer AJ, Ginn HM, Zhao Y, Duyvesteyn HME, Tuekprakhon A, Nutalai R, Wang B (2021). Screaton, Evidence of escape of SARS-CoV-2 variant B.1.351 from natural and vaccine-induced sera. Cell.

[22] Tas JM, Mesin L, Pasqual G, Targ S, Jacobsen JT, Mano YM, Chen CS, Weill JC, Reynaud CA, Browne EP, Meyer-Hermann M (2016). Visualizing antibody affinity maturation in germinal centers. Science.

[23] Kuraoka M, Schmidt AG, Nojima T, Feng F, Watanabe A, Kitamura D, Harrison SC, Kepler TB, Kelsoe G (2016). Complex Antigens Drive Permissive Clonal Selection in Germinal Centers. Immunity.

[24] Abe T, Sakaue-Sawano A, Kiyonari H, Shioi G, Inoue K, Horiuchi T, Nakao K, Miyawaki A, Aizawa S, Fujimori T (2013). Visualization of cell cycle in mouse embryos with Fucci2 reporter directed by Rosa26 promoter. Development.

[25] Ersching J, Efeyan A, Mesin L, Jacobsen JT, Pasqual G, Grabiner BC, Dominguez-Sola D, Sabatini DM, Victora GD (2017). Germinal Center Selection and Affinity Maturation Require Dynamic Regulation of mTORC1 Kinase. Immunity.

[26] Radtke D, Bannard O (2018). Expression of the Plasma Cell Transcriptional Regulator Blimp-1 by Dark Zone Germinal Center B Cells During Periods of Proliferation. Front Immunol.

[27] Hashimoto K, Joshi SK, Koni PA (2002). A conditional null allele of the major histocompatibility IA-beta chain gene. Genesis.

[28] Ventura A, Kirsch DG, McLaughlin ME, Tuveson DA, Grimm J, Lintault L, Newman J, Reczek EE, Weissleder R, Jacks T (2007). Restoration of p53 function leads to tumour regression in vivo. Nature.

[29] Bannard O, McGowan SJ, Ersching J, Ishido S, Victora GD, Shin JS, Cyster JG (2016). Ubiquitin-mediated fluctuations in MHC class II facilitate efficient germinal center B cell responses. J Exp Med.

[30] Pape KA, Catron DM, Itano AA, Jenkins MK (2007). The humoral immune response is initiated in lymph nodes by B cells that acquire soluble antigen directly in the follicles. Immunity.

[31] Suan D, Kräutler NJ, Maag JLV, Butt D, Bourne K, Hermes JR, Avery DT, Young C, Statham A, Elliott M, Dinger ME (2017). CCR6 Defines Memory B Cell Precursors in Mouse and Human Germinal Centers, Revealing Light-Zone Location and Predominant Low Antigen Affinity. Immunity.

[32] Wang Y, Shi J, Yan J, Xiao Z, Hou X, Lu P, Hou S, Mao T, Liu W, Ma Y, Zhang L (2017). Germinal-center development of memory B cells driven by IL-9 from follicular helper T cells. Nat Immunol.

[33] Laidlaw BJ, Schmidt TH, Green JA, Allen CD, Okada T, Cyster JG (2017). The Eph-related tyrosine kinase ligand Ephrin-B1 marks germinal center and memory precursor B cells. J Exp Med.

[34] Ge Q, Bai A, Shen CH, Eisen HN, Chen J (2003). CD4+ T-cell responses to self-peptide--MHC. Trends Immunol.

[35] Moran AE, Holzapfel KL, Xing Y, Cunningham NR, Maltzman JS, Punt J, Hogquist KA (2011). T cell receptor signal strength in Treg and iNKT cell development demonstrated by a novel fluorescent reporter mouse. J Exp Med.

[36] Paus D, Phan TG, Chan TD, Gardam S, Basten A, Brink R (2006). Antigen recognition strength regulates the choice between extrafollicular plasma cell and germinal center B cell differentiation. J Exp Med.

[37] Inoue T, Shinnakasu R, Ise W, Kawai C, Egawa T, Kurosaki T (2017). The transcription factor Foxo1 controls germinal center B cell proliferation in response to T cell help. J Exp Med.

[38] Luo W, Weisel F, Shlomchik MJ (2018). B Cell Receptor and CD40 Signaling Are Rewired for Synergistic Induction of the c-Myc Transcription Factor in Germinal Center B Cells. Immunity.

[39] Davidzohn N, Biram A, Stoler-Barak L, Grenov A, Dassa B, Shulman Z (2020). Syk degradation restrains plasma cell formation and promotes zonal transitions in germinal centers. J Exp Med.

[40] Shinnakasu R, Inoue T, Kometani K, Moriyama S, Adachi Y, Nakayama M, Takahashi Y, Fukuyama H, Okada T, Kurosaki T (2016). Regulated selection of germinal-center cells into the memory B cell compartment. Nat Immunol.

[41] Nakagawa R, Toboso-Navasa A, Schips M, Young G, Bhaw-Rosun L, Llorian-Sopena M, Chakravarty P, Sesay AK, Kassiotis G, Meyer-Hermann M, Calado DP (2021). Permissive selection followed by affinity-based proliferation of GC light zone B cells dictates cell fate and ensures clonal breadth. Proc Natl Acad Sci U S A.

[42] Zhang Y, Tech L, George LA, Acs A, Durrett RE, Hess H, Walker LSK, Tarlinton DM, Fletcher AL, Hauser AE, Toellner KM (2018). Plasma cell output from germinal centers is regulated by signals from Tfh and stromal cells. J Exp Med.

[43] Tze LE, Horikawa K, Domaschenz H, Howard DR, Roots CM, Rigby RJ, Way DA, Ohmura-Hoshino M, Ishido S, Andoniou CE, Degli-Esposti MA (2011). CD83 increases MHC II and CD86 on dendritic cells by opposing IL-10-driven MARCH1-mediated ubiquitination and degradation. J Exp Med.

[44] Krzyzak L, Seitz C, Urbat A, Hutzler S, Ostalecki C, Gläsner J, Hiergeist A, Gessner A, Winkler TH, Steinkasserer A (2016). CD83 Modulates B Cell Activation and Germinal Center Responses. J Immunol.

[45] Lam KP, Kühn R, Rajewsky K (1997). In vivo ablation of surface immunoglobulin on mature B cells by inducible gene targeting results in rapid cell death. Cell.

[46] Shulman Z, Gitlin AD, Weinstein JS, Lainez B, Esplugues E, Flavell RA, Craft JE, Nussenzweig MC (2014). Dynamic signaling by T follicular helper cells during germinal center B cell selection. Science.

[47] Finney J, Yeh CH, Kelsoe G, Kuraoka M (2018). Germinal center responses to complex antigens. Immunol Rev.

[48] Dominguez-Sola D, Victora GD, Ying CY, Phan RT, Saito M, Nussenzweig MC, Dalla-Favera R (2012). The proto-oncogene MYC is required for selection in the germinal center and cyclic reentry. Nat Immunol.

[49] Calado DP, Sasaki Y, Godinho SA, Pellerin A, Köchert K, Sleckman BP, de Alborán IM, Janz M, Rodig S, Rajewsky K (2012). The cell-cycle regulator c-Myc is essential for the formation and maintenance of germinal centers. Nat Immunol.

[50] Meyer-Hermann M (2021). A molecular theory of germinal center B cell selection and division. Cell Rep.

[51] Kräutler NJ, Suan D, Butt D, Bourne K, Hermes JR, Chan TD, Sundling C, Kaplan W, Schofield P, Jackson J, Basten A (2017). Differentiation of germinal center B cells into plasma cells is initiated by high-affinity antigen and completed by Tfh cells. J Exp Med.

[52] Young C, Brink R (2021). The unique biology of germinal center B cells. Immunity.

[53] Dominguez-Sola D, Kung J, Holmes AB, Wells VA, Mo T, Basso K, Dalla-Favera R (2015). The FOXO1 Transcription Factor Instructs the Germinal Center Dark Zone Program. Immunity.

[54] Sander S, Chu VT, Yasuda T, Franklin A, Graf R, Calado DP, Li S, Imami K, Selbach M, Di Virgilio M, Bullinger L, Rajewsky K (2015). PI3 Kinase and FOXO1 Transcription Factor Activity Differentially Control B Cells in the Germinal Center Light and Dark Zones. Immunity.

[55] Ortega-Molina A, Deleyto-Seldas N, Carreras J, Sanz A, Lebrero-Fernández C, Menéndez C, Vandenberg A, Fernández-Ruiz B, Marín-Arraiza L, de la Calle Arregui C, Belén Plata-Gómez A (2019). Oncogenic Rag GTPase signaling enhances B cell activation and drives follicular lymphoma sensitive to pharmacological inhibition of mTOR. Nat Metab.

[56] Turner JS, Benet ZL, Grigorova I (2017). Transiently antigen primed B cells can generate multiple subsets of memory cells. PLoS One.

[57] Zhang Y, Meyer-Hermann M, George LA, Figge MT, Khan M, Goodall M, Young SP, Reynolds A, Falciani F, Waisman A, Notley CA (2013). Germinal center B cells govern their own fate via antibody feedback. J Exp Med.

[58] Duan L, Liu D, Chen H, Mintz MA, Chou MY, Kotov DI, Xu Y, An J, Laidlaw BJ, Cyster JG (2021). Follicular dendritic cells restrict interleukin-4 availability in germinal centers and foster memory B cell generation. Immunity.

[59] Phan TG, Amesbury M, Gardam S, Crosbie J, Hasbold J, Hodgkin PD, Basten A, Brink R (2003). B cell receptor-independent stimuli trigger immunoglobulin (Ig) class switch recombination and production of IgG autoantibodies by anergic self-reactive B cells. J Exp Med.

[60] Robbiani DF, Bothmer A, Callen E, Reina-San-Martin B, Dorsett Y, Difilippantonio S, Bolland DJ, Chen HT, Corcoran AE, Nussenzweig A, Nussenzweig MC (2008). AID is required for the chromosomal breaks in c-myc that lead to c-myc/IgH translocations. Cell.

[61] Buch T, Heppner FL, Tertilt C, Heinen TJ, Kremer M, Wunderlich FT, Jung S, Waisman A (2005). A Cre-inducible diphtheria toxin receptor mediates cell lineage ablation after toxin administration. Nat Methods.

[62] Hobeika E, Levit-Zerdoun E, Anastasopoulou V, Pohlmeyer R, Altmeier S, Alsadeq A, Dobenecker MW, Pelanda R, Reth M (2015). CD19 and BAFF-R can signal to promote B-cell survival in the absence of Syk. Embo j.

[63] Moran I, Nguyen A, Khoo WH, Butt D, Bourne K, Young C, Hermes JR, Biro M, Gracie G, Ma CS, Munier CML (2018). Memory B cells are reactivated in subcapsular proliferative foci of lymph nodes. Nat Commun.

[64] Barnden MJ, Allison J, Heath WR, Carbone FR (1998). Defective TCR expression in transgenic mice constructed using cDNA-based alpha- and beta-chain genes under the control of heterologous regulatory elements. Immunol Cell Biol.

[65] Mombaerts P, Clarke AR, Rudnicki MA, Iacomini J, Itohara S, Lafaille JJ, Wang L, Ichikawa Y, Jaenisch R, Hooper ML (1992). Mutations in T-cell antigen receptor genes alpha and beta block thymocyte development at different stages. Nature.

[66] Itohara S, Mombaerts P, Lafaille J, Iacomini J, Nelson A, Clarke AR, Hooper ML, Farr A, Tonegawa S (1993). T cell receptor delta gene mutant mice: independent generation of alpha beta T cells and programmed rearrangements of gamma delta TCR genes. Cell.

[67] Lee PP, Fitzpatrick DR, Beard C, Jessup HK, Lehar S, Makar KW, Pérez-Melgosa M, Sweetser MT, Schlissel MS, Nguyen S, Cherry SR (2001). A critical role for Dnmt1 and DNA methylation in T cell development, function, and survival. Immunity.

