# Supplementary material for: Competition for refuelling rather than cyclic re-entry initiation evident in germinal centers
Source: Sci Immunol. Author manuscript; Available in PMC 2023 May 3. (PMC7614495; doi:10.1126/sciimmunol.abm0775)
Supplement: Supplementary Material [file EMS174788-supplement-Supplementary_Material.pdf]

Supplementary Materials

Figure S1. (Related to Figure 1) Validation of Fucci2 model.

Figure S2. (Related to Figure 3) Validation of the MHCII deletion approach and extension of findings related to cyclic re-entry initiation by MHCII-deleted cells.

Figure S3. (Related to Figure 4) Evidence that acute competition between LZ cells may not restrict cyclic re-entry initiation.

Figure S4. (Related to Figure 6) Extension of data related to BATF induction and role of cell-cell competition.

Figure S5. (Related to Figure 7) High and low affinity cells initiate cyclic at different time points post-mitosis.

Figure S6. Association between cell cycle stage and BCR affinity in LZs and DZs.

Table S1. Raw data excel file

Supplementary Materials

Figure S1. (Related to Figure 1) Validation of Fucci2 model.

Figure S2. (Related to Figure 3) Validation of the MHCII deletion approach and extension of findings related to cyclic re-entry initiation by MHCII-deleted cells.

Figure S3. (Related to Figure 4) Evidence that acute competition between LZ cells may not restrict cyclic re-entry initiation.

Figure S4. (Related to Figure 6) Extension of data related to BATF induction and role of cell-cell competition.

Figure S5. (Related to Figure 7) High and low affinity cells initiate cyclic at different time points post-mitosis.

Figure S6. Association between cell cycle stage and BCR affinity in LZs and DZs.

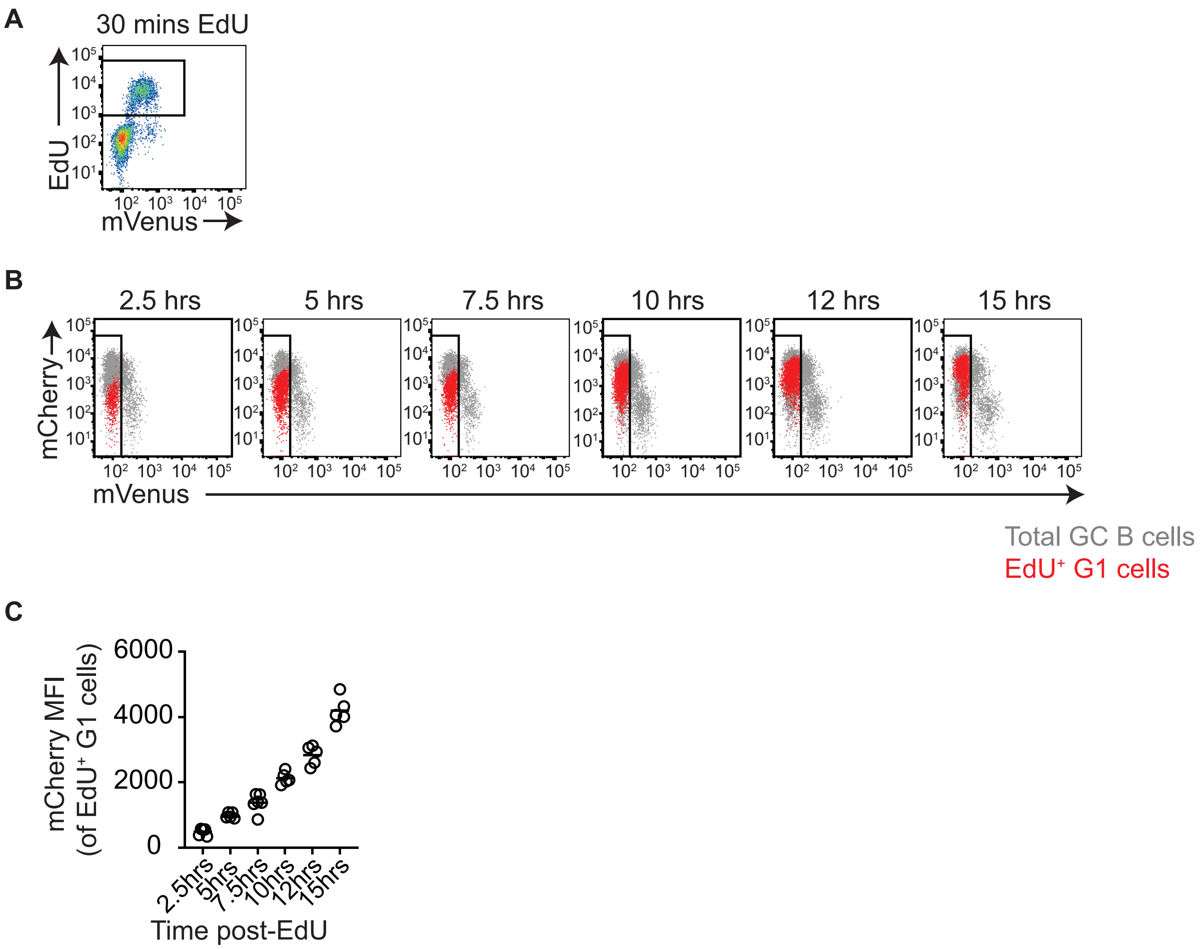

Figure S1. (Related to Figure 1) Validation of Fucci2 model.

SW_{HEL} x Fucci2 CD45.1 B cells were transferred to WT CD45.2 hosts that were subsequently immunised with HEL^{3X}-SRBCs/LPS. Mice received single EdU injections to tag and chase S phase cells at the indicated time points prior to analysis. SW_{HEL} GC B cells (CD45.1 IgD^{low} $CD95^{+}$ $GL7^{+}$) were analysed on day 8. (A) Association between EdU incorporation and mVenus was assessed following a 30 mins EdU treatment. (B) Mice receiving EdU >30mins also received a single BrdU injection 20 mins before analysis to assist in gating G1 cells (i.e., $BrdU^{-}$, compensating for losses in mVenus resolution from fixation). mCherry fluorescent intensities by EdU^{+} G1 ($mVenus^{-}BrdU^{-}$) (red) and total GC B cells (grey) were compared at the indicated time points post-EdU. (C) Quantitation of results from (B). The slightly reduced intensity spreads in A, B, compared to Fig. 1, result from fixation. A, B are representative, and C or pooled, from 2 experiments, with each point representing a mouse. MFIs are normalised across experiments.

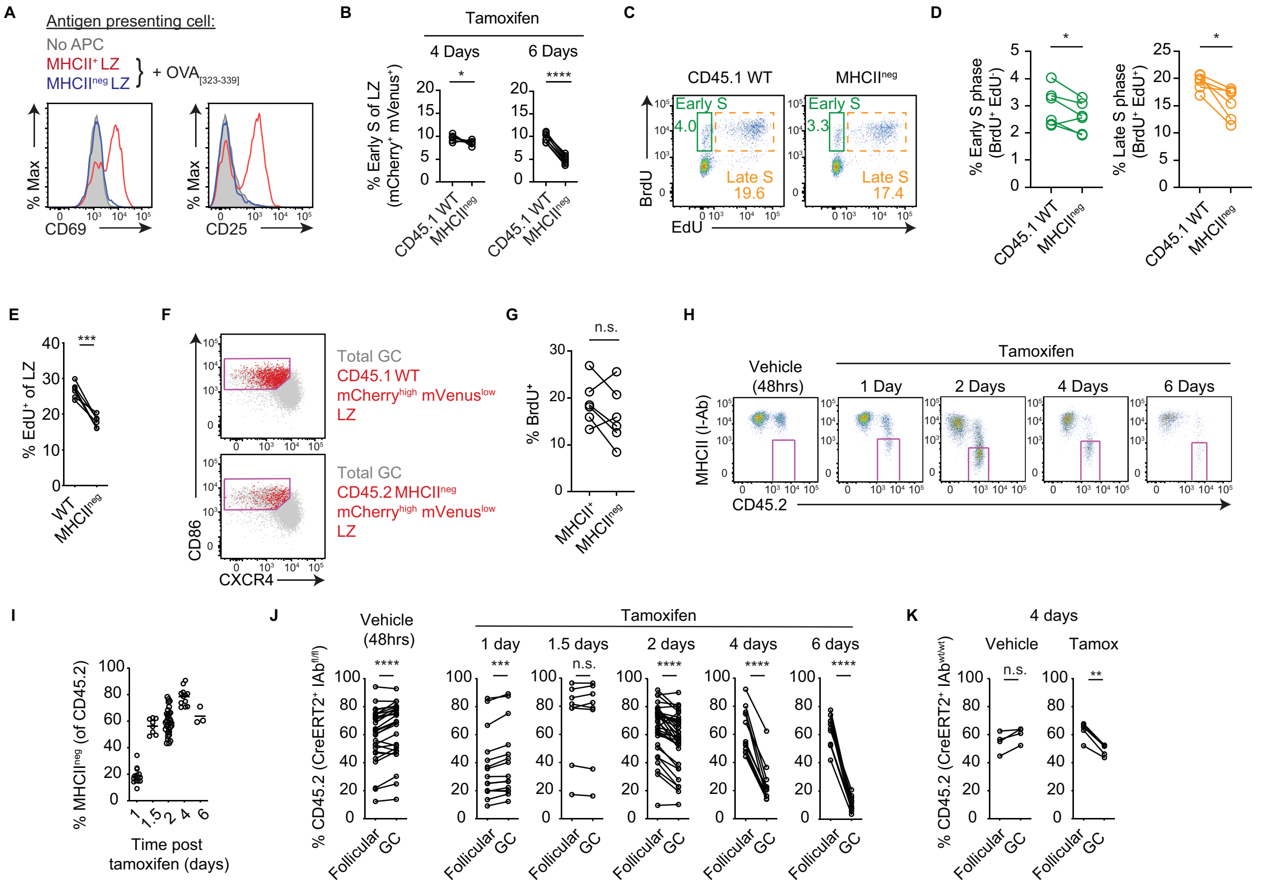
A
Antigen presenting cell:
No APC
MHCII+ LZ
MHCIIneg LZ
+ OVA[323-339]
% Max
CD69
CD25
B
Tamoxifen
4 Days
6 Days
% Early S of LZ (mCherry+ mVenus+)
CD45.1 WT
MHCIIneg
C
CD45.1 WT
MHCIIneg
Early S
Late S
4.0
19.6
3.3
17.4
BrdU
EdU
D
% Early S phase (BrdU+ EdU-)
% Late S phase (BrdU+ EdU+)
E
% EdU+ of LZ
WT
MHCIIneg
F
Total GC
CD45.1 WT
mCherryhigh mVenuslow
LZ
Total GC
CD45.2 MHCIIneg
mCherryhigh mVenuslow
LZ
CD86
CXCR4
G
n.s.
% BrdU+
MHCII+
MHCIIneg
H
Vehicle (48hrs)
Tamoxifen
1 Day
2 Days
4 Days
6 Days
MHCII (I-Ab)
CD45.2
I
% MHCIIneg (of CD45.2)
Time post tamoxifen (days)
J
Vehicle (48hrs)
Tamoxifen
1 day
1.5 days
2 days
4 days
6 days
% CD45.2 (CreERT2+ IAbfl/fl)
Follicular
GC
K
4 days
Vehicle
Tamox
% CD45.2 (CreERT2+ IAbwt/wt)

Figure S2. (Related to Figure 3) Validation of the MHCII deletion approach and extension of findings related to cyclic re-entry initiation by MHCII-deleted cells.

CreERT2^{+} MHCII$^{fl/fl}$ CD45.2/ WT CD45.1 BM chimeric mice (or non-chimeric CreERT2^{+} MHCII$^{fl/fl}$ mice in A, and (40% WT Fucci2 CD45.1: 60% CreERT2^{+} MHCII$^{fl/fl}$ Fucci2 CD45.2 chimeric mice in B)) were immunised with SRBCs, with tamoxifen (or vehicle) treatment on day 7. (A) One day post-tamoxifen treatment, FACS sorted MHCII^{+} and MHCII-deleted LZ cells were co-cultured overnight with purified OT-II CD4^{+} T cells plus exogenous peptide, then activation marker induction on T cells was assessed. (B) Extension of results in Fig 3C-D. Frequencies of early S phase (mVenus^{+} mCherry^{+}) LZ cells at day 4 and day 6 time points post-tamoxifen. (C, D) Mixed BM chimeras as in (A) were analysed on day 8 post-immunisation, 48hrs after tamoxifen treatment. The mice received single EdU and BrdU injections 100 mins and 40 mins before analysis. (C, D) Frequencies of early S (green) and late S (dashed orange) cells among WT and MHCII-deleted LZ populations. (E) Similar experiments but immunising with NP-KLH/alum (i.p.), with tamoxifen and EdU treatments (identifying S phase cells) given 2.5 days and 30 mins, respectively, before analysis on day 12. (F) Related to Figure 3C, 48hrs time point. WT and MHCII-deleted early S phase LZ cells (Red) were overlaid with the total GC population (grey). (G) SW$_{HEL}$ *Cd79a*-CreERT2^{+} MHCII$^{fl/fl}$ B cells, together with OT-II T cells, were transferred into *Tcrb/d*$^{-/-}$ hosts that were subsequently immunised with HEL-OVA/Ribi (i.p.). Mice received tamoxifen and BrdU injections 43hrs and 30mins before analysis on day 8. Frequencies of S phase LZ cells are shown. (H, I) Frequencies of MHCII-deleted cells within LZs in mixed BM chimeras, showing various time points day 6 post-tamoxifen treatment. (J) Frequencies of CD45.2 CreERT2^{+} MHCII$^{fl/fl}$ cells within naïve follicular and GC populations. (K) The same assessment was made for control BM chimeras containing CreERT2^{+} MHCII$^{wt/wt}$ cells. FACS plots and summary graphs in A, C, F, H, and B, D, E, G, I, J, K are representative of, or pooled from (respectively), 2-10 independent experiments (per time point) each containing 3-5 mice per condition. K summarizes a single experiment. Data points in graphs each represent single mice. Lines join populations from individual mice. Analysis, paired two-tailed Student's t test. *$p < 0.05$, **$p < 0.01$, ***$p < 0.001$., ****$p<0.0001$.

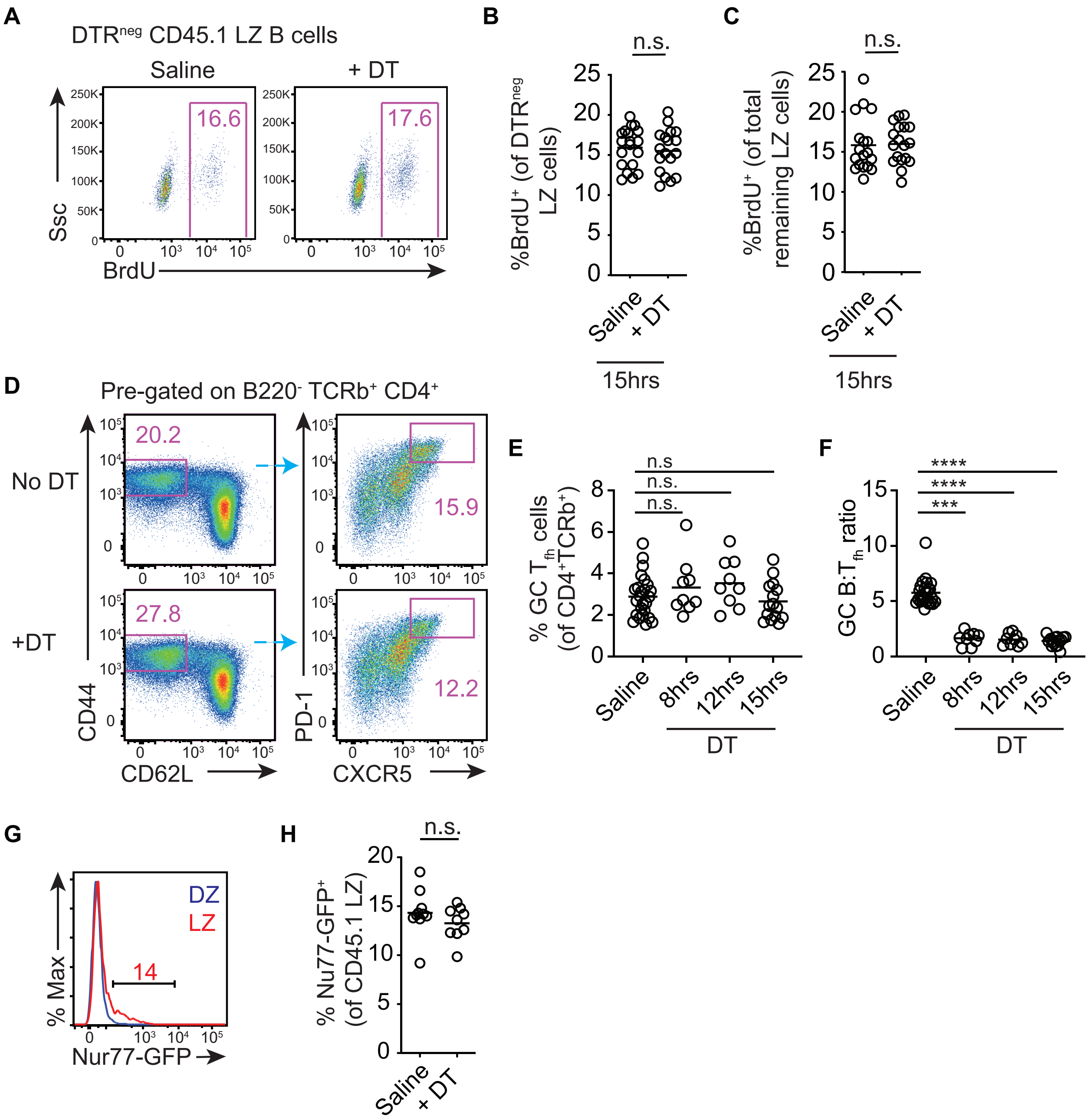
A
DTRneg CD45.1 LZ B cells
Saline
+ DT
16.6
17.6
Ssc
BrdU
B
n.s.
%BrdU+ (of DTRneg LZ cells)
Saline
+ DT
15hrs
C
n.s.
%BrdU+ (of total remaining LZ cells)
Saline
+ DT
15hrs
D
Pre-gated on B220- TCRb+ CD4+
No DT
+DT
20.2
27.8
15.9
12.2
CD44
CD62L
PD-1
CXCR5
E
n.s
n.s.
n.s.
% GC Tfh cells (of CD4+TCRb+)
Saline
8hrs
12hrs
15hrs
DT
F
****
****
***
GC B:Tfh ratio
Saline
8hrs
12hrs
15hrs
DT
G
DZ
LZ
14
% Max
Nur77-GFP
H
n.s.
% Nu77-GFP+ (of CD45.1 LZ)
Saline
+ DT

Figure S3. (Related to Figure 4) Evidence that acute competition between LZ cells may not restrict cyclic re-entry initiation.

(A-C) BM chimeric mice containing ~80:20 mixes of AID-DTR and WT cells were immunised with SRBCs, then treated with DT (or saline) and BrdU 15 hrs and 30 mins before analysis on day 8, respectively. (A) BrdU incorporation by CD45.1 (DTR^{-ve}) LZ cells, (B) and summary of pooled data. (C) A similar analysis was performed on all remaining LZ cells (WT and remaining DTR^{+} cells). (D, E) Frequencies of GC T_{fh} cells were determined. (F) GC B:T_{fh} ratios at indicated time points. (G, H) Similar partial B cell ablation experiments were performed with mixed BM chimeras containing Nur77-GFP CD45.1 cells (~80:20 mixes in favour of DTR^{+} cells). Summaries in B, C, E, F, H are pooled data from 2-6 independent experiments with 2-4 mice/condition/experiment. FACS plots are representative data. Analysis, (B, C, H) unpaired two-tailed Student's t test, (E, F) Kruskal-Wallis one-way ANOVA with multiple comparisons, ***$p < 0.001$, ****$p<0.0001$.

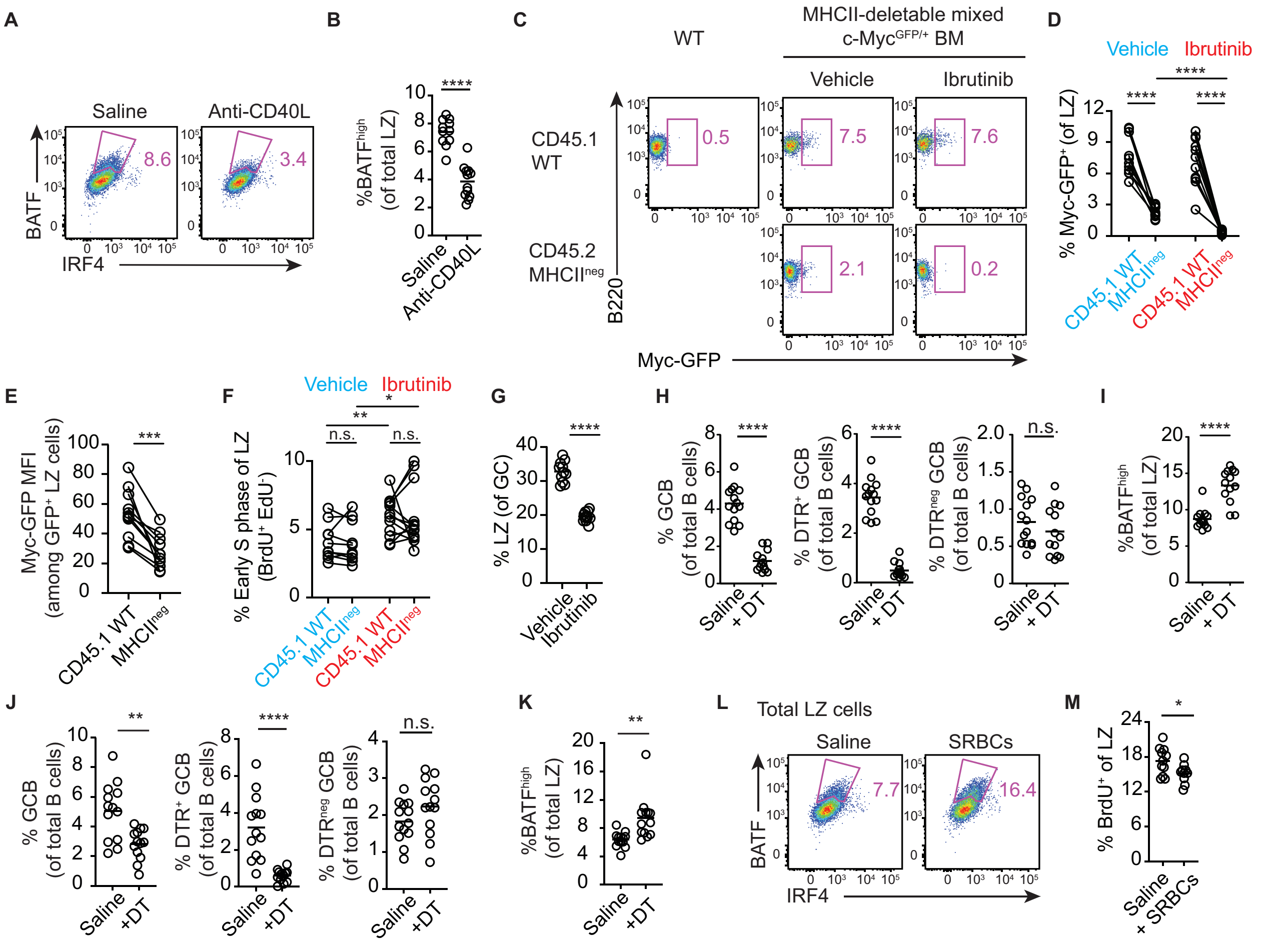

A
Saline
Anti-CD40L
8.6
3.4
BATF
IRF4
B
****
%BATF^high (of total LZ)
Saline
Anti-CD40L
C
WT
MHCII-deletable mixed c-Myc^GFP/+ BM
Vehicle
Ibrutinib
CD45.1 WT
CD45.2 MHCII^neg
0.5
7.5
7.6
2.1
0.2
B220
Myc-GFP
D
Vehicle
Ibrutinib
****
% Myc-GFP⁺ (of LZ)
CD45.1 WT
MHCII^neg
CD45.1 WT
MHCII^neg
E
***
Myc-GFP MFI (among GFP⁺ LZ cells)
CD45.1 WT
MHCII^neg
F
Vehicle
Ibrutinib
*
**
n.s.
n.s.
% Early S phase of LZ (BrdU⁺ EdU⁻)
CD45.1 WT
MHCII^neg
CD45.1 WT
MHCII^neg
G
****
% LZ (of GC)
Vehicle
Ibrutinib
H
****
% GCB (of total B cells)
% DTR⁺ GCB (of total B cells)
n.s.
% DTR^neg GCB (of total B cells)
Saline
+ DT
I
****
%BATF^high (of total LZ)
Saline
+ DT
J
**
% GCB (of total B cells)
****
% DTR⁺ GCB (of total B cells)
n.s.
% DTR^neg GCB (of total B cells)
Saline
+DT
K
**
%BATF^high (of total LZ)
Saline
+DT
L
Total LZ cells
Saline
SRBCs
7.7
16.4
BATF
IRF4
M
*
% BrdU⁺ of LZ
Saline
+ SRBCs

Figure S4. (Related to Figure 6) Extension of data related to BATF induction and role of cell-cell competition.

(A, B) WT mice were immunised with SRBCs, then treated with anti-CD40L antibody or saline 24hrs prior to analysis on day 8. Splenic LZ GC B (IgD^{low} CD95^{+} GL7^{+} CXCR4^{low} CD86^{+}) cells were analysed for BATF induction. (C-F) BM chimeras containing mixes of CreERT2^{+} MHCII$^{fl/fl}$ c-Myc$^{GFP/+}$ CD45.2 and MHCII$^{+/+}$ c-Myc$^{GFP/+}$ CD45.1 cells were immunised with SRBCs, treated with tamoxifen on day 7 and then either ibrutinib or vehicle for 7hrs prior to analysis on day 8 (24hrs post-tamoxifen). EdU and BrdU treatments were also given at 100 mins and 40 mins before analysis, to identify early S phase cells. (C) Representative FACs plots and (D) enumeration of Myc-GFP^{+} cell frequencies among CXCR4^{low} CD86^{+} GC B cells (IgD^{low} CD95^{+} GL7^{+}). (E) Myc-GFP expression levels (MFI) on a per cell basis, among Myc-GFP^{+} gated cells. Results presented as MFI minus Myc-GFP^{-} MFI (WT) to allow experiment pooling. (F) Frequencies of LZ cells in early S phase and, (G) frequencies of LZ cells within total GC. (H-I) Extension of results from Fig. 6D; (H) Frequencies of total GC B cells (left), DTR^{+} GC B cells (middle) and DTR^{-ve} GC B cells (right). (I) Frequencies of total LZ cells (irrespective of genotype) expressing high BATF levels. (J, K) Similar experiments using BM chimeras with less severe mixing ratios (~55:45 AID-DTR^{+}:WT). (L) Representative FACs plot of LZ cells from Fig. 6F, 24hrs after additional SRBC injection; (M) the mice also received 30min BrdU treatments and the frequency of S phase cells within LZs was determined. Same control mice used in Fig. S4A and 6F. Analysis, unpaired two-tailed Student's t test except comparisons within mice in D, E, F, which were paired. *$p < 0.05$, **$p < 0.01$, ***$p < 0.001$, ****$p<0.0001$.

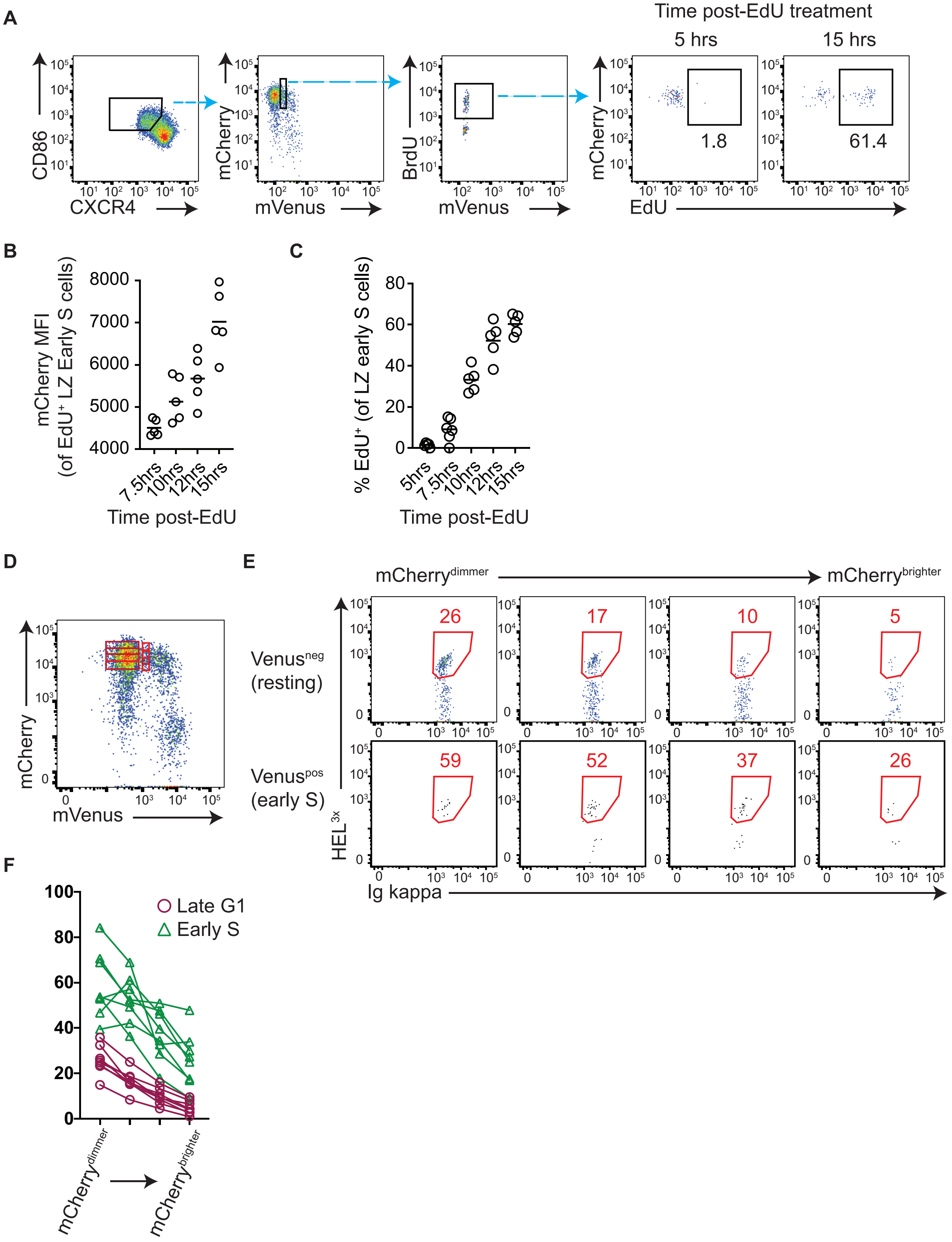

A
Time post-EdU treatment
5 hrs
15 hrs
CD86
CXCR4
mCherry
mVenus
BrdU
mVenus
mCherry
EdU
1.8
61.4
B
mCherry MFI
(of EdU+ LZ Early S cells)
8000
7000
6000
5000
4000
7.5hrs
10hrs
12hrs
15hrs
Time post-EdU
C
% EdU+ (of LZ early S cells)
80
60
40
20
0
5hrs
7.5hrs
10hrs
12hrs
15hrs
Time post-EdU
D
mCherry
mVenus
E
mCherrydimmer
mCherrybrighter
Venusneg
(resting)
Venuspos
(early S)
HEL3x
Ig kappa
26
17
10
5
59
52
37
26
F
100
80
60
40
20
0
Late G1
Early S
mCherrydimmer
mCherrybrighter

Figure S5. (Related to Figure 7) High and low affinity cells initiate cyclic at different time points post-mitosis.

SW_{HEL} Fucci2 CD45.1 B cells were adoptively transferred to WT CD45.2 hosts that were subsequently immunised with HEL^{3X}-SRBCs/LPS. Analysis of GC B cells (CD45.1 IgD^{low} $CD95^{+}$ $GL7^{+}$) was performed on day 8. (A) Mice received single EdU injections at the indicated times prior to analysis to tag and chase S phase. Mice also received BrdU injections 20 mins before tissue harvest (compensating for mVenus resolution losses caused by fixation, to improve S phase identification). Early S phase LZ B cells were gated (left) and EdU incorporation measured (right). (B) mCherry MFIs of EdU^{+} early S phase LZ cells at the indicated times, and (C), the frequencies of EdU^{+} cells. (D-F) In separate experiments (using CD45.1 IgD^{low} $GL7^{+}$ or CD45.1 $GL7^{+}$ GC gates), late G1 and early S phase cells were divided into 4 subgroups based on mCherry levels. (E) Frequencies of HEL^{3X}-binding cells were determined for each subset, means shown. (F) Pooled results from 8 mice in 2 experiments, with lines joining subsets from individual mice. Results in (A) are representative, and (B, C) pooled, from two experiments containing 2-3 mice/time point, with each data point from a single animal. MFIs in (B) are normalized across experiments.

A

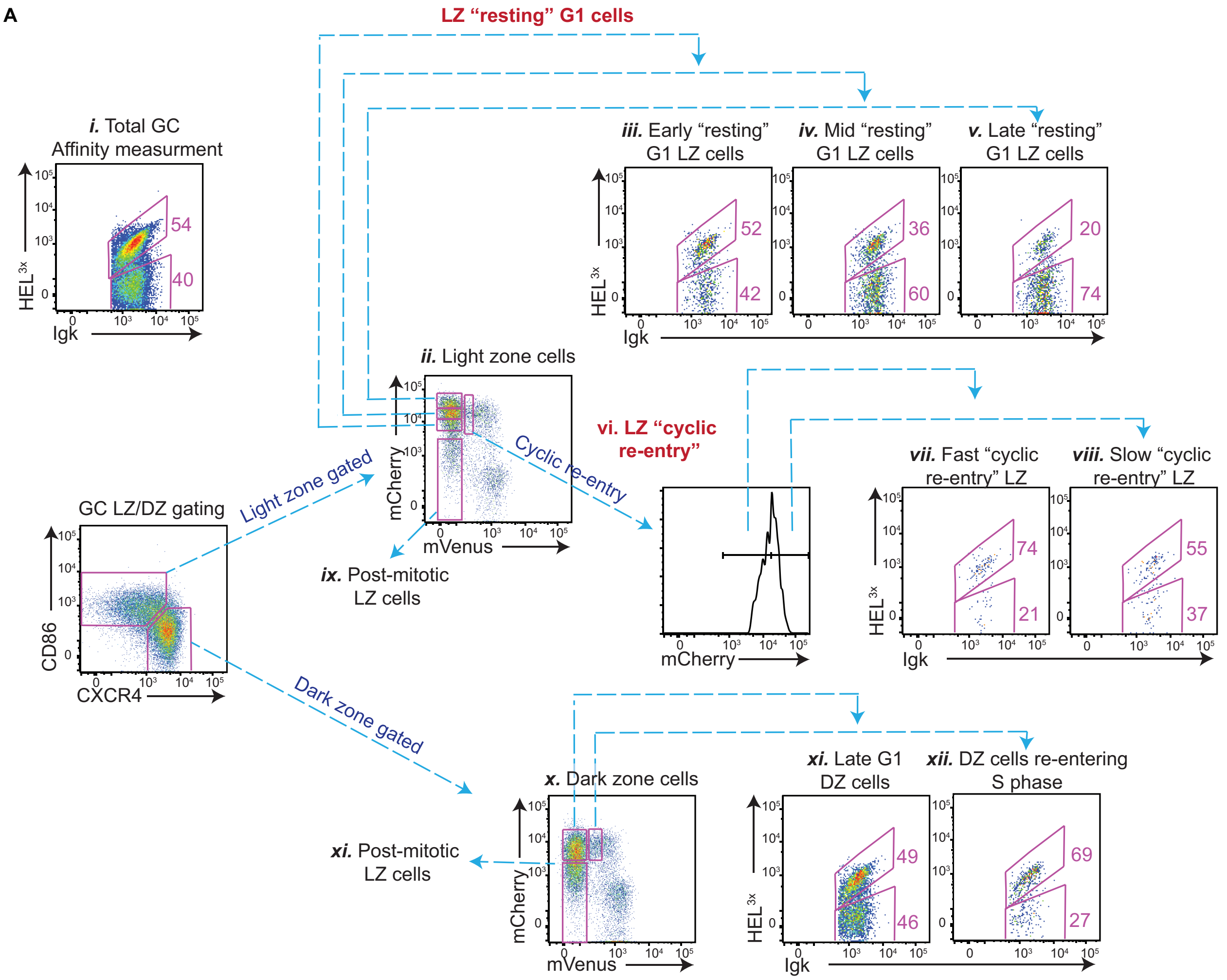

LZ "resting" G1 cells
i. Total GC
Affinity measurment
54
40
HEL 3x
Igk
iii. Early "resting"
G1 LZ cells
iv. Mid "resting"
G1 LZ cells
v. Late "resting"
G1 LZ cells
52
42
36
60
20
74
ii. Light zone cells
mCherry
mVenus
vi. LZ "cyclic
re-entry"
Cyclic re-entry
Light zone gated
GC LZ/DZ gating
CD86
CXCR4
ix. Post-mitotic
LZ cells
vii. Fast "cyclic
re-entry" LZ
viii. Slow "cyclic
re-entry" LZ
74
21
55
37
Dark zone gated
x. Dark zone cells
xi. Post-mitotic
LZ cells
xi. Late G1
DZ cells
xii. DZ cells re-entering
S phase
49
46
69
27

B

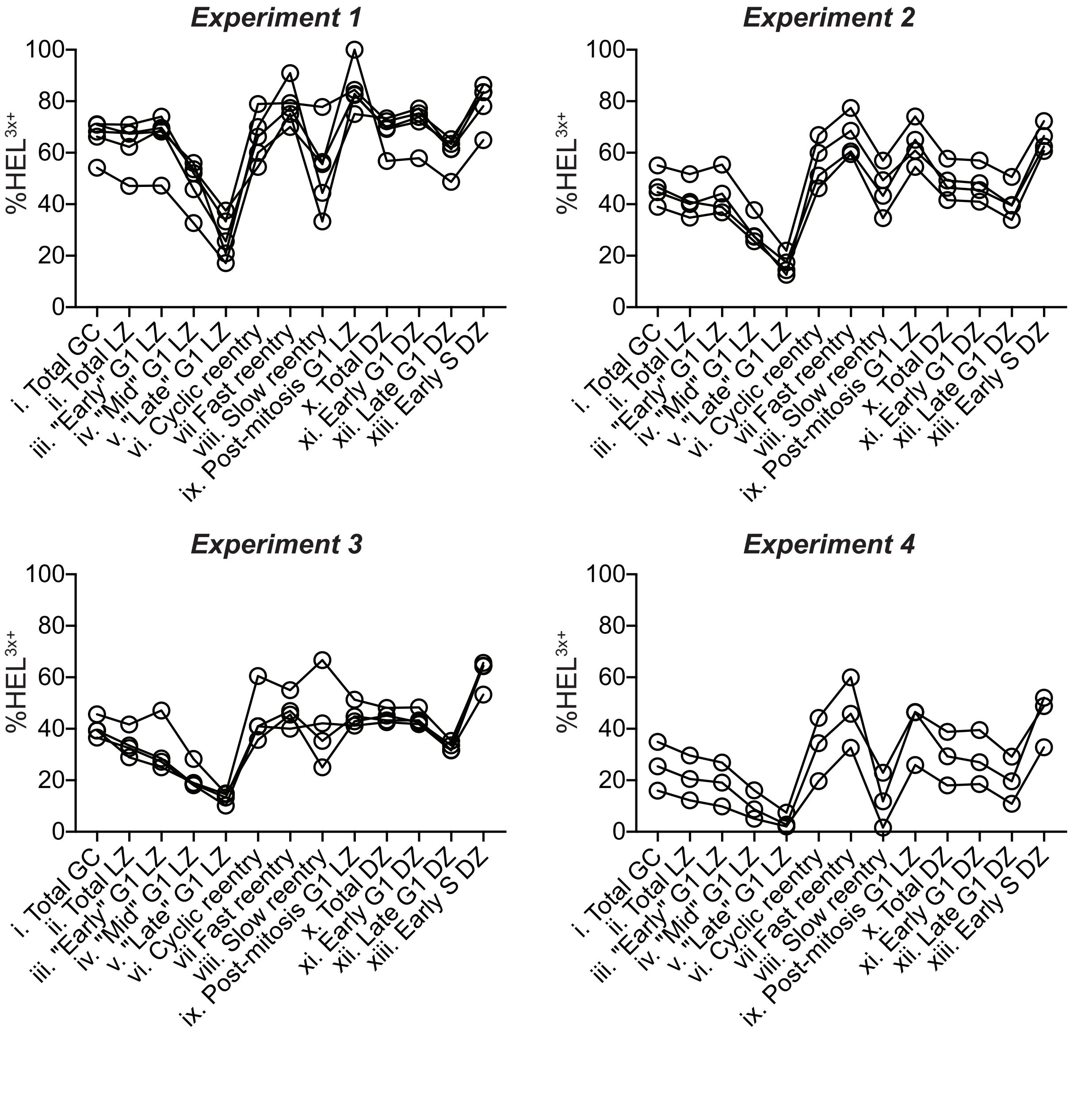
Experiment 1
Experiment 2
Experiment 3
Experiment 4
%HEL^{3x+}
0
20
40
60
80
100
i. Total GC
ii. Total LZ
iii. "Early" G1 LZ
iv. "Mid" G1 LZ
v. "Late" G1 LZ
vi. Cyclic reentry
vii Fast reentry
viii. Slow reentry
ix. Post-mitosis G1 LZ
x. Total DZ
xi. Early G1 DZ
xii. Late G1 DZ
xiii. Early S DZ

Figure S6. Association between cell cycle stage and BCR affinity in LZs and DZs.

SW_{HEL} Fucci2 CD45.1 B cells were adoptively transferred to CD45.2 WT hosts that were subsequently immunised with HEL^{3X}-SRBCs/LPS. Analysis of GC B cells (CD45.1 IgD^{low} $CD95^{+}$ $GL7^{+}$) was performed on day 8. LZ and DZ subsets were further gated based upon their cell cycle stage. Frequencies of HEL^{3x}-binding cells were identified for each subset. (B) Summary of results from 4 experiments each containing multiple mice. Lines join populations from individual mice. i-xii on (A) indicate same populations on (B).
